# Thymidine-Inosine Dimer Building Block for Reversible Modification of Synthetic Oligonucleotides

**DOI:** 10.3390/molecules30183769

**Published:** 2025-09-17

**Authors:** Natalia A. Kolganova, Irina V. Varizhuk, Andrey A. Stomakhin, Marat M. Khisamov, Pavel N. Solyev, Sergei A. Surzhikov, Edward N. Timofeev

**Affiliations:** W.A. Engelhardt Institute of Molecular Biology, Russian Academy of Sciences, Vavilov St. 32, Moscow 119991, Russia; natha@yandex.ru (N.A.K.); irina.varizhuk@gmail.com (I.V.V.); stomstom@hotmail.com (A.A.S.); hisamovmaratag@mail.ru (M.M.K.); solyev@gmail.com (P.N.S.); ssergey77@mail.ru (S.A.S.)

**Keywords:** oligonucleotides, reversible modification, click chemistry, oxidative cleavage

## Abstract

Modification of synthetic oligonucleotides and DNA is widely used in many applications in the life sciences. However, in most cases, modified DNA cannot be restored to its native state. Here, we report the preparation of a thymidine-inosine dimer building block (TID) for oligonucleotide synthesis. The TID modification supports the functionalization of synthetic oligonucleotides, which can later be removed to restore the DNA strand to its native state. The TID unit allows for a wide spectrum of postsynthetic modifications of oligonucleotides through click chemistry, including conjugation with fluorescent tags and small molecules, preparation of branched oligonucleotide scaffolds, and anchoring to a solid support. Due to the modification of the thymine base, the TID unit reduces the stability of the DNA duplex. We found that the negative effect of internal TID modification on duplex stability does not exceed the same for a single base mismatch. As long as the TID modification is present in the DNA strand, it disrupts its natural functionality. The “caging” effect of TID in the template strand with respect to DNA polymerase was demonstrated in primer extension experiments. Traceless removal of the temporary functional group occurs through oxidative cleavage of the inosine subunit, resulting in the formation of a native DNA strand with the thymine base left at the cleavage site. An anthracene-modified dodecamer oligonucleotide and a branched oligonucleotide scaffold were used to study the cleavage of the reporter group or the oligonucleotide side strand, respectively. It was shown that aqueous tetramethylguanidine efficiently cleaves the oxidized inosine subunit of TID at 37 °C, forming the native DNA strand.

## 1. Introduction

Currently, modified synthetic oligonucleotides have found numerous applications in nucleic acid therapeutics, diagnostics, bio- and nanotechnology, and molecular biology [1,2,3]. The growing demand for modified synthetic oligonucleotides and, in general, modified nucleic acids is associated with their increased resistance to biodegradation, as well as with the specific structural and functional features of modified nucleic acids [4,5,6]. The ease of chemical oligonucleotide synthesis and the wide range of available nucleoside and nonnucleoside building blocks provide high level of flexibility in programming the functionality, structural characteristics, and nuclease resistance of modified oligonucleotides. However, these advantages are associated with limitations regarding the native functionality of nucleic acids. Modified nucleic acids are rarely suitable substrates for numerous nucleic acid enzymes. Therefore, removable modification of a synthetic oligonucleotide for temporary functionality is desirable when the native characteristics of nucleic acids need to be restored at a later stage.

Significant progress has been made in the field of removable modifications of nucleic acids for temporary blocking of nucleic acid functions. Temporary blocking of native nucleic acid functionality has been implemented within the “cage-decage” concept for native or synthetic DNA and RNA [7,8,9,10]. In these studies, much of the focus has been on the development of biocompatible methods for removing the “caging” groups. Generally, the idea of cleavable linkers is widely used in bioorganic chemistry [11,12,13,14]. The described methods for the traceless recovery of native nucleic acids from their conjugates or modified state are based largely on photo-induced cleavage, action of chemical stimuli, and enzymatic reactions. Additionally, heat-removable “caging” groups have been proposed for thermolytic release of phosphorylated oligonucleotides [15,16]. This method has been implemented in hot-start PCR applications [15]. Since the primary goal of these studies was the “caging” functionality, little attention was paid to expanding the functional diversity of the cleavable group.

**Figure 1:**
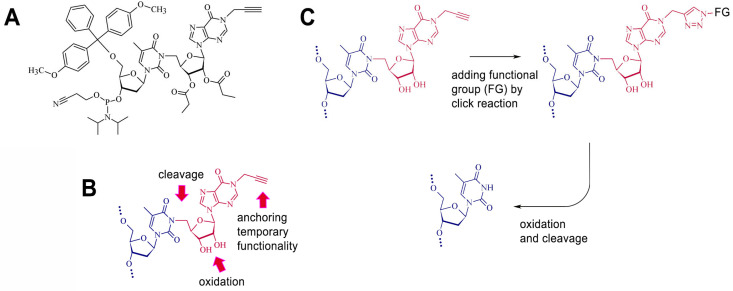
(**A**) TID phosphoramidite building block. (**B**) Functionality of TID subunits. (**C**) Adding and cleavage of a click-compatible transient functional group.

In the present study, we describe the synthesis of a novel alkyne-substituted thymidine-inosine dimer (TID) phosphoramidite for the reversible functionalization of synthetic oligonucleotides via click chemistry (Figure 1). This modification is incorporated into synthetic oligonucleotides using standard phosphoramidite chemistry and remains stable during deprotection procedure. A wide selection of functional azides can be used for further modification of the inosine subunit via click chemistry. Traceless removal of the modified residue is achieved through a combination of periodate oxidation of the sugar residue in the inosine subunit and subsequent elimination of the oxidized residue upon exposure to a strong base.

## 2. Results and Discussion

### 2.1. Design and Synthesis of the Cleavable Unit

The TID unit design supports a wide range of postsynthetic DNA modifications through click chemistry, i.e., bioconjugation, labeling, and anchoring to a solid support. In our case, the cleavage of the functional group is initiated by selective oxidation of the vicinal 2′,3′-diol in the inosine subunit of TID and proceeds through elimination of the oxidized sugar moiety. In designing of the cleavable unit, we selected the thymine base for attachment of the temporary functional group due to the simple and high-yield alkylation of its N3 position [17,18]. It has been shown that the N3 position of thymidine can be reversibly blocked by a cyanoethyl protecting group, which is cleaved under basic conditions via an elimination mechanism [19]. To generate suitable intermediates for the same type of cleavage, we used highly selective periodate oxidation of the vicinal diol fragment in the inosine subunit of the TID.

**Figure 2:**
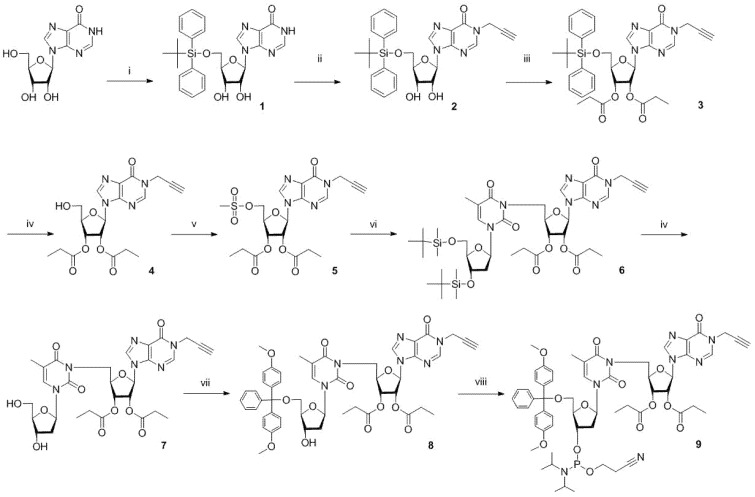
Synthesis of TID phosphoramidite **8**: (i) tBu(Ph)_2_SiCl, Im, Py; (ii) propargyl bromide, DBU, MeCN; (iii) propionic anhydride, Py; (iv) triethylamine trihydrofluoride, Et_3_N, THF; (v) MsCl, Et_3_N, DCM; (vi) 3′,5′-di-O-t-butyldimethylsilyl thymidine, DBU, MeCN; (vii) DMTrCl, Py; (viii) 2-cyanoethyl N,N,N′,N′-tetraisopropylphosphorodiamidite, Py, tetrazole, MeCN.

TID phosphoramidite, a building block for oligonucleotide synthesis, was prepared starting from inosine in nine-step synthesis (Figure 2). The synthesis strategy was optimized to prevent the migration of the 2′ and 3′ acyl groups, which was a major concern. Selective protection of the 5′ hydroxyl group of inosine was followed by alkylation at the N1 position with propargyl bromide. The secondary hydroxyl groups were then acylated with propionic anhydride. Removal of the silyl protecting group was carried out using triethylamine buffered (HF)_3_-Et_3_N. This step was not complicated by the migration of the acyl group, as was the case when using tetrabutylammonium fluoride. Activation of the 5′ hydroxyl group of the inosine subunit and coupling to a protected thymidine afforded silylated TID precursor. Deprotection of the thymidine subunit, addition of a 4,4′-dimethoxytrityl protecting group to the 5′ hydroxyl group, and phosphitylation yielded the TID cyanoethyl phosphoramidite.

### 2.2. Functional, Biophysical, and Biochemical Characterization of TID-Modified Oligonucleotides

#### 2.2.1. Synthesis of TID-Modified Oligonucleotides, Their Small-Molecule Conjugates, and Branched Oligonucleotides

**Figure 3:**
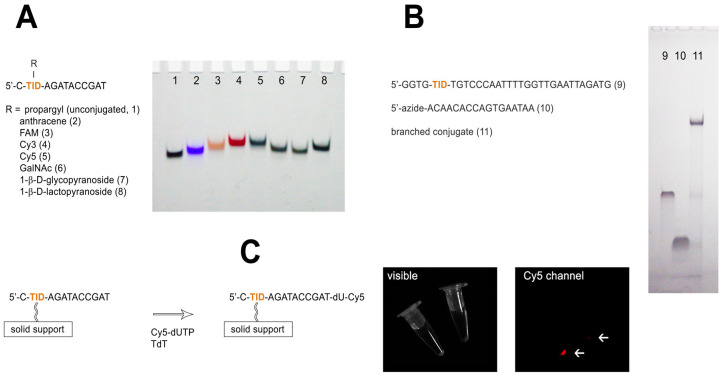
Three types of reversible oligonucleotide functionalization by TID modification. (**A**) Analysis of small molecules conjugates of TID-modified dodecanucleotide by denaturing PAGE. The bands were visualized by UV shadowing. (**B**) Analysis of the branched oligonucleotide scaffold by denaturing PAGE. (**C**) Extension of the support-bound dodecamer with a fluorescent dUTP derivative in the presence of terminal deoxynucleotide transferase (TdT). Arrows indicate the fluorescence of the sample and the negative control (without support-bound dodecamer).

To verify the ability of the TID unit to support a wide range of modifications, we used a short dodecamer sequence 5′-C-TID-AGATACCGAT. The TID unit was added to the DNA sequence during automated oligonucleotide synthesis using phosphoramidite **9**. It is worth noting that TID residue may be used at any desired position of the synthetic oligonucleotide. To ensure efficient modification, the coupling time was increased from 25 to 60 s. Standard ammonia deprotection at 55 °C for 6 h and purification by reverse phase HPLC yielded the TID-modified oligonucleotide. The presence of the modified unit in the dodecamer sequence was confirmed by MALDI mass-spectrometry (Supplementary Figure S1). In the next step, a series of oligonucleotide TID-conjugates were prepared using Cu-TBTA and azide derivatives of reporter molecules, mono- and disaccharides (Figure 3A). In addition to fluorescent reporter groups widely used for the modification of synthetic oligonucleotides, carbohydrate oligonucleotide conjugates have attracted much attention in recent years in the context of cellular delivery of oligonucleotide therapeutics [20,21]. We conjugated TID-modified dodecamer with azide derivatives of anthracene, fluorescein, cyanine dyes, N-acetyl galactosamine (GalNAc), 1-β-D-glycopyranose, and 1-β-D-lactopyranose (Supplementary Figure S2). Analysis of dodecamer conjugates by denaturing polyacrylamide gel electrophoresis (PAGE) confirmed the specific functionalization of the oligonucleotide (Figure 3A).

Branched oligonucleotide scaffolds with cleavable side strands may be of notable interest for DNA nanotechnology due to their multiplexed functionality and ability for selective disassembly [22]. The TID unit supports the postsynthetic assembly of branched oligonucleotide scaffolds through conjugation with azide-modified oligonucleotides. To demonstrate the utility of the TID modification in the preparation of branched oligonucleotide scaffolds, we conjugated a 31-nt modified oligonucleotide with a 5′ azide derivative of the oligonucleotide ACAACACCAGTGAATAA (Figure 3B). The latter was prepared by the treatment of the respective 5′-aminooligonucleotide with an azide-PEG6-NHS linker (Lumiprobe, Moscow, Russia). For the conjugation, we used oligonucleotides in a 1:1 ratio. The formation of a longer branched oligonucleotide scaffold was confirmed by PAGE (Figure 3B) under denaturing condition.

#### 2.2.2. Anchoring of TID-Modified Dodecamer to a Solid Support

Click chemistry offers a rapid and convenient technique to anchor synthetic oligonucleotides to a solid support [23]. Immobilized oligonucleotides find widespread application in life science, primarily as immobilized hybridization probes or primers. TID modification perfectly suits the click-based immobilization method. Furthermore, it offers the possibility of recovering a support-bound oligonucleotide probe or an enzymatically extended DNA strand in its native state. As a simple model, we immobilized TID-modified dodecamer to an azide-functionalized controlled pore glass (CPG) solid support (Figure 3C and Supplementary Figure S3). For this purpose, amino-derivatized Hybrid CPG (Prime Synthesis Inc., Aston, PA, USA) was treated with an azide-PEG6-NHS linker (Lumiprobe, Moscow, Russia). Click-immobilization of the TID-dodecamer was performed in aqueous DMF. The immobilization yield (68–74%) was estimated by quantifying the unmodified oligomer that was recovered by oxidative cleavage of the bound probe (Supplementary Figure S4). The average loading density of the bound probe was 0.96 pmol/mg. The CPG-bound dodecamer was functional in enzymatic reactions, as evidenced by tagging its 3′ end with Cy5-dUTP using terminal deoxynucleotide transferase (Figure 3C).

#### 2.2.3. “Caging” Effect of the TID Modification in the Primer Extension Reaction

**Figure 4:**
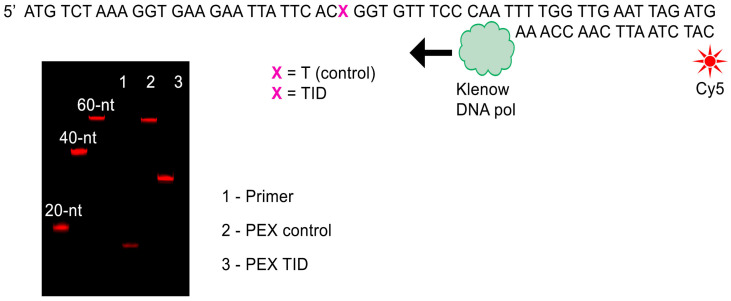
Primer extension arrest by TID modification. Fluorescent extension products derived from modified and unmodified template strands were resolved by denaturing PAGE. Cy5-labeled 20-, 40-, and 60-nt oligomers were used as controls.

The presence of a bulky substituent at position N3 of the thymine base in TID interferes with the ability of a modified DNA strand to be a substrate for nucleic acids enzymes. We examined the substrate properties of the TID-modified DNA strand in a primer extension reaction. TID-modified and unmodified 57-nt oligonucleotides were synthesized and used as templates in the primer extension reaction (Figure 4). A Cy5-labeled 17-nt primer was annealed to either natural or modified template and extended by Klenow DNA polymerase at 37 °C in the presence of four natural dNTPs. Primer extension products were resolved by denaturing PAGE and visualized by Cy5 fluorescence. The presence of TID residue in the template strand induced a polymerization arrest at the site of modification. Modified residue does not support enzymatic DNA polymerization until present in the template strand and can be used to temporarily block the template strand functionality.

#### 2.2.4. Effect of Internal TID Modification on DNA Duplex Stability

Modification of the thymine base in the TID unit prevents the formation of the canonical TA base pair in duplex DNA. To estimate the effect of the TID modification on DNA duplex stability, we used modified and unmodified dodecameric oligonucleotides and the four complementary strands, 5′-ATCGGTATCTYG (where Y = A, G, C, or T). The thermodynamic stability of short DNA duplexes was studied in UV melting experiments at 260 nm in 1 M NaCl and 10 mM sodium phosphate buffer (pH 7.5). The data presented in Table 1 show that the TID residue induced a decrease in the *T*_m_ value by 3.5 °C relative to the perfect duplex, i.e., TID/A vs. T/A base pair. The loss of stability of the modified duplexes with the other three variants of the complementary DNA strand varied in the range 4.1–5.2 °C. Interestingly, base mismatches formed by natural nucleotides showed somewhat higher destabilization effect. The average value of Δ*T*_m_ for the four TID-modified duplexes was 4.4 °C vs. 5.1 °C for imperfect unmodified duplexes. Thus, the internal TID modification appears to have a less negative impact on duplex stability than a single internal base mismatch.

It should be noted that the observed effect of modification on duplex stability is characteristic of the unconjugated TID unit. Further click conjugation through the TID propargyl group can notably affect duplex stability.

### 2.3. Oxidative Cleavage of the Inosine Subunit and Recovery of the Native DNA Strand

**Figure 5:**
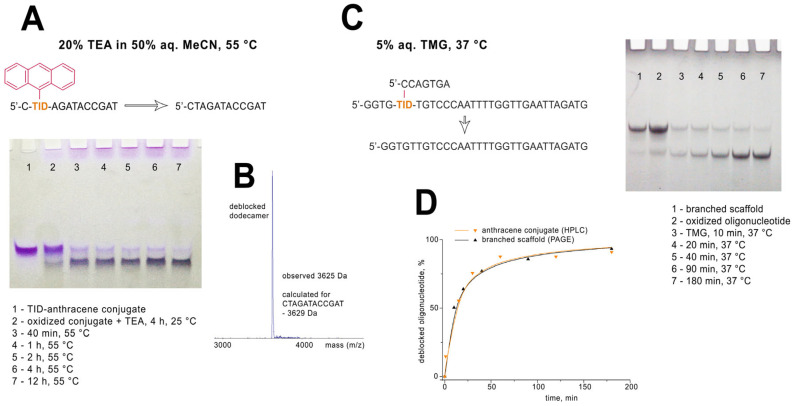
Oxidative cleavage of TID oligonucleotide derivatives. (**A**) Recovery of the native dodecanucleotide from its TID-anthracene conjugate using 20% TEA in aqueous acetonitrile at 55 °C. (**B**) Post-cleavage analysis of the dodecanucleotide by MALDI MS. (**C**) Oxidative cleavage of the short side strand of the branched oligonucleotide scaffold with 5% aqueous TMG at 37 °C. (**D**) Cleavage kinetics for the two oligonucleotide models (TMG, 37 °C). The standard error did not exceed 3%.

The oxidative cleavage of the modified unit of the synthetic DNA strand was studied using either the anthracene dodecanucleotide conjugate or the branched oligonucleotide scaffold (Figure 5). With regard to the dodecanucleotide model, the presence of a large hydrophobic anthracene tag made it possible monitoring of the oligonucleotide transformations using different analytical techniques, i.e., MALDI mass spectrometry, gel electrophoresis, and HPLC. The oxidation of the inosine subunit in the TID-anthracene oligonucleotide conjugate was induced by an aqueous NaIO_4_ or Et_3_N-HIO_4_. The latter reagent turned out to be more convenient since the excess oxidizing agent could be easily removed by the precipitation of the oligonucleotide derivative. The oxidation step can be as short as few minutes at room temperature when using 100 mM periodate solution.

Efficient cleavage of the oxidized inosine unit requires exposure to a strong base, preferably at elevated temperatures. Under neutral conditions, the oxidized form of the TID-anthracene oligonucleotide was rather stable and showed a well-defined single band in PAGE analysis. We found that the oxidized anthracene conjugate was stable for at least 1 h at 25 °C in 100 mM aqueous Tris-Base, 100 mM K_2_CO_3_, or 5% aqueous N,N-dimethylaminopyridine. Heating the oxidized oligonucleotide in NEB ThermoPol^®^ PCR buffer (pH 8.8) at 72 °C for 30 min did not result in cleavage of the anthracene. At 94 °C in the same buffer, only minor cleavage was detected after 30 min of heating.

Concentrated aqueous ammonia or triethylamine in aqueous acetonitrile induced efficient cleavage of the oxidized inosine-anthracene subunit at 55 °C (Figure 5A). We found that the native DNA strand recovered almost quantitatively (>95%) after 12 h of heating with 20% triethylamine in 50% aqueous acetonitrile at 55 °C. In the case of ammonia-induced deprotection, an oligonucleotide intermediate containing a cyclic morpholino-type inosine subunit was initially formed, as confirmed by MALDI-MS analysis (Supplementary Figure S5). The most efficient elimination was observed in 5% aqueous 1,1,3,3-tetramethylguanidine (TMG) at 37 °C. Fast conversion (>90%) of the oxidized anthracene conjugate to native DNA strand was observed within the first 3 h as evidenced by PAGE and HPLC analyses. Complete cleavage after 12 h of TMG exposure was confirmed by MALDI analysis (Figure 5B).

Another model oligonucleotide for studying oxidative cleavage was a branched oligonucleotide scaffold with a 7-nucleotide side strand. It was prepared by conjugating the corresponding azide derivative of the heptamer oligonucleotide with the TID-modified 31-mer as described above. Cleavage of the short side strand in 5% TMG at 37 °C was monitored by denaturing PAGE (Figure 5C). Quantitative analysis of the PAGE bands showed cleavage kinetics very similar to those observed for the anthracene conjugate when its cleavage products were analyzed by reversed-phase HPLC (Figure 5D).

Our results demonstrate that TID is a versatile chemical tool for a wide range of click-compatible postsynthetic oligonucleotide modifications with the optional possibility of restoring native DNA functionality. In the context of spatiotemporal control of nucleic acid functionality, TID modification falls into a family of stimuli-responsive protecting groups that block nucleic acid functions until a controlled event triggers the recovery of a specific oligonucleotide or DNA function. However, unlike the typical “caging” modification, the TID unit can function in reverse, first adding new functionality and subsequently removing it via oxidative cleavage. Furthermore, the TID modification features the expanded functionality of a typical “caging” group, including precise positioning in a sequence, compatibility with chemical oligonucleotide synthesis, chemical stability, a wide selection of post-synthetic modifications, and traceless elimination through oxidative cleavage. These characteristics make TID a promising tool for numerous applications in diagnostics or nanotechnology. On the other hand, the use of toxic periodate salts and TMG in the cleavage step prevents immediate use of TID modification for in vivo applications. The oxidized form of TID has been shown to be quite stable and can form conjugates with amino-containing compounds, yielding morpholino-type intermediates. This peculiarity can potentially be used for efficient delivery of oxidized TID-oligonucleotides to cells while protecting the reactive dialdehyde group. Further studies are needed to verify whether oxidized inosine subunit or its derivatives can be efficiently eliminated in the intracellular environment.

## 3. Conclusions

Here we report a synthetic approach for the traceless removal of an oligonucleotide modification for reversible functionalization. The incorporation of the TID residue into a synthetic DNA strand provides the possibility for further modification with a wide range of functional groups. The latter can be optionally removed to restore the native DNA sequence. Oxidative cleavage of the TID modification restores the natural DNA strand with the thymidine base remaining at the cleavage site. To ensure maximum flexibility of this approach, the tethering of the functional group proceeds through the propargyl substituent of the TID residue via orthogonal click chemistry. The range of applications for TID modification covers a wide variety of manipulations, including the recovery of the natural DNA strand from covalent conjugates with reporter groups or other small molecules, as well as with other biomolecules, and the recovery of the support-bound DNA. The cleavage chemistry relies on the highly specific periodate oxidation of the vicinal diol group and subsequent elimination of the oxidized fragment. The TID unit can be preserved under any condition that the DNA itself or the temporary functionality can withstand until the oxidation step is performed. With regard to biocompatibility of the TID modification, further studies are required to optimize the modified unit and identify mild cleavage conditions for in vivo applications.

## 4. Experimental Section

### 4.1. General

Chemical reagents and solvents were purchased from various commercial suppliers and used without further purification. Standard reagents for automated oligonucleotide synthesis were purchased from Glen Research (Sterling, VA, USA). Fluorescent dye azides, N_3_-PEG6-NHS linker, Cy5-dUTP, and Cu(II)-TBTA complex (10 mM in DMSO) were purchased from Lumiprobe (Moscow, Russia). GalNAc azide was purchased from BLD Pharmatech Ltd. (Shanghai, China). Azide derivatives of 1-β-D-glycopyranose and 1-β-D-lactopyranose were purchased from Merck KGAA (Darmstadt, Germany). Klenow DNA polymerase was purchased from New England Biolab (Ipswich, MA, USA). Terminal deoxynucleotide transferase (TdT) was from Thermo Fisher Scientific (Waltham, MA, USA). Aqueous triethylammonium periodate was prepared from NaIO_4_ solution by ion-exchange using Dowex 50WX8 in the triethylammonium form. Thin layer chromatography (TLC) was carried out using Kieselgel 260 F aluminum sheets (Merck, Darmstadt, Germany) and visualized by UV absorption or stained with 2% H_2_SO_4_ in EtOH followed by heating. The following solvent mixtures were used for TLC: DCM/EtOH 9:1 (A); DCM/hexane/EtOH 9:10:1 (B); and ethyl acetate/hexane 3:1 (C). Column chromatography was performed using silica gel (particle size 0.06–0.2 mm, Sigma-Aldrich, St. Louis, MO, USA). Nuclear magnetic resonance spectra were recorded with a Bruker AVANCE II 300 spectrometer (Bruker, Billerica, MA, USA). The residual solvent signals were used as references and the chemical shifts were converted to the TMS scale (DMSO-d6: δH = 2.50 ppm, δC = 39.51 ppm). High-resolution mass spectra were recorded on a Bruker micrOTOF-Q II device by electrospray ionization mass spectrometry (ESI-MS). Measurements were carried out in positive ion mode; samples were injected into the mass-spectrometer chamber from an HPLC system Agilent 1260 (Agilent, Santa-Clara, CA, USA). The MALDI mass spectra were acquired using a 4800 Plus mass spectrometer (AB Sciex, Framingham, MA, USA) in linear mode. Spectra were recorded for positive ions. Before analysis, the samples were treated with Dowex 50WX8 in ammonium form.

### 4.2. Synthesis

#### 4.2.1. 5′-O-Tert-Butyldiphenylsilyl Inosine (**1**)

Imidazole (0.5 g, 7.35 mmol) and *t*-butyldiphenylsilyl chloride (2 g, 7.35 mmol) were added to a stirred suspension of inosine (1.92 g, 7.16 mmol) in a mixture of pyridine (10 mL) and DMF (2 mL). The mixture was heated to 40 °C and stirred at 25 °C for 24 h. Pyridine was evaporated under reduced pressure. The residue was diluted with DCM (150 mL) and washed successively with saturated aqueous NaHCO_3_ (100 mL) and water (100 mL). The organic layer was dried over anhydrous Na_2_SO_4_ and concentrated in vacuum. The residue (5 mL) was precipitated with diethyl ether (50 mL) yielding 2.76 g (76%) of the title compound. R_f_ (A): 0.4. ^1^H-NMR (300.1 MHz, DMSO-*d*_6_): δ = 12.3 (brs, 1H, NH), 8.21 (s, 1H, H-2), 7.99 (s, 1H, H-8), 7.67–7.56 and 7.51–7.32 (m, 10H, aromatic), 5.92 (d, 1H, *J*_1′,2′_ = 4.8 Hz, H1′), 5.58 (d, 1H, *J*_2′-OH_ = 5.6 Hz, 2′-OH), 5.25 (d, 1H, *J*_3′-OH_ = 5.6 Hz, 3′-OH), 4.55 (dd, 1H, *J*_2′,3′_ = 5.0 Hz, H2′), 4.30 (dd, 1H, *J*_3′,4′_ = 4.6 Hz, H3′), 4.04 (dd, 1H, *J*_4′,5′a_ = 3.6 Hz, *J*_4′,5′b_ = 4.9 Hz, H4′), 3.93 (dd, 1H, *J*_5′a′,5′b_ = −11.4 Hz, H5′a), 3.79 (dd, 1H, H5′b), 0.99 (s, 9H, tBu). ^13^C-NMR (75.5 MHz, DMSO-*d*_6_): 157.00 (C6), 148.66 (C4), 146.26 (C2), 138.97 (C8), 135.57, 135.48, 133.25, 133.09, 130.34, 128.34, and 128.30 (CH Ph), 124.98 (C5), 88.13 (C1′), 84.87 (C4′), 74.07 (C2′), 70.23 (C3′), 64.32 (C5′), 27.10 (CH_3_ tBu), 19.25 (C tBu). HRMS (ESI) calculated [M+H]^+^ for C_26_H_30_N_4_O_5_Si: 507.2058, found: 507.2061.

#### 4.2.2. N^1^-Propargyl-5′-O-Tert-Butyldiphenylsilyl Inosine (**2**)

DBU (2.4 mL, 16 mmol) and propargyl bromide (80% soln. in toluene, 2 mL, 16 mmol) were added to a stirred solution of compound **1** (2.7 g, 5.3 mmol) in 10 mL of a mixture of MeCN-EtOH (3:1 *v*/*v*). The reaction mixture was heated to 40 °C for 5 min and stirred at room temperature for an additional 30 min. Then, the solvents were evaporated under reduced pressure. The residue was dissolved in 150 mL of DCM and washed with water (100 mL). The organic layer was dried over anhydrous Na_2_SO_4_ and concentrated in vacuum. The residue was dissolved in 5 mL of DCM, precipitated with diethyl ether (50 mL), and purified by column chromatography on silica gel using gradient elution from DCM/hexane/EtOH 10:10:1 to DCM/hexane/EtOH 10:5:1 yielding 2.37 g (82%) of compound **2**. R_f_ (A): 0.85. ^1^H-NMR (300.1 MHz, DMSO-d6): δ = 8.42 (s, 1H, H-2), 8.26 (s, 1H, H-8), 7.66–7.58 and 7.50–7.32 (m, 10H, aromatic), 5.91 (d, 1H, *J*_1′,2′_ = 4.8 Hz, H1′), 5.58 (d, 1H, *J*_2′-OH_ = 5.7 Hz, 2′-OH), 5.26 (d, 1H, *J*_3′-OH_ = 5.6 Hz, 3′-OH), 4.84 (d, 2H, *J_N_*_-CH2, *≡*CH_ = 2.4 Hz, N-CH_2_), 4.57 (dd, 1H, *J*_2′,3′_ = 5.1 Hz, H2′), 4.31 (dd, 1H, *J*_3′,4′_ = 4.8 Hz, H3′), 4.05 (ddd, 1H, *J*_4′,5′a_ = 3.7 Hz, *J*_4′,5′b_ = 4.9 Hz, H4′), 3.92 (dd, 1H, *J*_5′a′,5′b_ = −11.3 Hz, H5′a), 3.79 (dd, 1H, H5′b), 3.43 (t, 1H, ≡CH), 0.98 (s, 9H, tBu). ^13^C-NMR (75.5 MHz, DMSO-d6): 155.65 (C6), 148.25 (C2), 147.81 (C4), 139.89 (C8), 135.57, 135.48, 133.23, 133.09, 130.35, 128.33, and 128.28 (CH Ph), 124.25 (C5), 88.28 (C1′), 84.97 (C4′), 79.20 (C≡CH), 76.07 (C≡CH), 74.01 (C2′), 70.23 (C3′), 64.28 (C5′), 35.38 (N-CH_2_ propargyl), 27.09 (CH_3_ tBu), 19.25 (C tBu). HRMS (ESI) calculated [M+H]^+^ for C_29_H_32_N_4_O_5_Si: 545.2215, found: 545.2211.

#### 4.2.3. N^1^-Propargyl-2′,3′-di-O-Propionyl, 5′-O-Tert-Butyldiphenylsilyl Inosine (**3**)

Compound **2** (2.3 g, 4.2 mmol) in pyridine (20 mL) was treated with propionic anhydride (2.7 mL, 21.2 mmol) for 12 h at 25 C. Then, methanol (5 mL) was added to the reaction mixture. After 30 min the mixture was concentrated in vacuum, dissolved in 200 mL of DCM, and washed successively with saturated aqueous NaHCO_3_ (100 mL) and water (100 mL). The organic layer was dried over anhydrous Na_2_SO_4_ and concentrated under reduced pressure. The residue was purified by column chromatography on silica gel using gradient elution from DCM/hexane 1:1 to DCM/hexane/EtOH 7:5:1, yielding 2.24 g (81%) of white sticky foam. R_f_ (B): 0.65. ^1^H-NMR (300.1 MHz, DMSO-*d*_6_): δ = 8.38 (s, 1H, H-2), 8.27 (s, 1H, H-8), 7.63–7.55 and 7.50–7.32 (m, 10H, aromatic), 6.20 (d, 1H, *J*_1′,2′_ = 5.3 Hz, H1′), 5.98 (dd, 1H, *J*_2′,3′_ = 5.8 Hz, H2′), 5.75 (dd, 1H, *J*_3′,4′_ = 4.8 Hz, H3′), 4.91–4.77 (m, 2H, N-CH_2_), 4.29 (dd, 1H, *J*_4′,5′a_ = 3.7 Hz, *J*_4′,5′b_ = 4.5 Hz, H4′), 3.96 (dd, 1H, *J*_5′a′,5′b_ = −11.5 Hz, H5′a), 3.85 (dd, 1H, H5′b), 3.42 (t, 1H, *J_N_*_-CH2*,* ≡CH_ = 2.5 Hz, ≡CH), 2.45–2.28 (m, 4H, CH_2_ propionyl), 1.08 (t, 3H, *J*_C3,CH3_ = 7.5 Hz, CH_3_ propionyl), 1.03–0.95 (m, 12H, CH_3_ propionyl, tBu). ^13^C-NMR (75.5 MHz, DMSO-*d*_6_): 173.07, 172.94 (O-C=O), 155.53 (C6), 148.46 (C2), 147.54 (C4), 140.36 (C8), 135.53, 135.49, 132.92, 132.77, 130.42, 128.32, and 128.29 (CH Ph), 124.51 (C5), 86.30 (C1′), 82.67 (C4′), 79.01 (C≡CH), 76.22 (C≡CH), 72.87 (C2′), 70.11 (C3′), 63.44 (C5′), 35.46 (N-CH_2_ propargyl), 27.09 (CH_2_ propionyl), 26.97 (C tBu), 26.92 (CH_2_ propionyl), 19.20 (C tBu), 9.37, 9.20 (CH_3_ propionyl). HRMS (ESI) calculated [M+H]^+^ for C_35_H_40_N_4_O_7_Si: 657.2739, found: 657.2740.

#### 4.2.4. N^1^-Propargyl-2′,3′-di-O-Propionyl Inosine (**4**)

To a solution of compound **3** (2.2 g, 3.4 mmol) in THF (5 mL) triethylamine (2.5 mL) and triethylamine trihydrofluoride (2.5 mL) were added. After 4 h, the reaction mixture was diluted with DCM (200 mL) and washed with 10% aqueous sodium chloride (100 mL). The organic layer was dried over anhydrous Na_2_SO_4_ and concentrated under reduced pressure. The residue was purified by column chromatography on silica gel using gradient elution from DCM/hexane/EtOH 10:10:1 to DCM/hexane/EtOH 5:5:1 yielding 1.0 g (71%) of compound **5**. R_f_ (B): 0.45. ^1^H-NMR (300.1 MHz, DMSO-*d*_6_): δ = 8.53 (s, 1H, H-2), 8.41 (s, 1H, H-8), 6.17 (d, 1H, *J*_1′,2′_ = 6.5 Hz, H1′), 5.85 (dd, 1H, *J*_2′,3′_ = 5.7 Hz, H2′), 5.52 (dd, 1H, *J*_3′,4′_ = 3.1 Hz, H3′), 5.33 (dd, 1H, *J*_5′a,OH_ = 5.2 Hz, *J*_5′b,OH_ = 5.8 Hz, 5′-OH), 4.85 (d, 2H, *J_N_*_-CH2, ≡CH_ = 2.5 Hz, N-CH_2_), 4.24 (dd, 1H, *J*_4′,5′a_ = 3.8 Hz, *J*_4′,5′b_ = 3.7 Hz, H4′), 3.74 (ddd, 1H, *J*_5′a′,5′b_ = −12.2 Hz, H5′a), 3.64 (ddd, 1H, H5′b), 3.40 (t, 1H, ≡CH), 2.48–2.37 (m, 2H, CH_2_ propionyl), 2.35–2.22 (m, 2H, CH_2_ propionyl), 1.08 (t, 3H, *J*_CH2, -CH3_ = 7.5 Hz, CH_3_ propionyl), 0.96 (t, 3H, *J*_CH2, -CH3_ = 7.5 Hz, CH_3_ propionyl). ^13^C-NMR (75.5 MHz, DMSO-*d*_6_): 173.24, 172.86 (O-C=O x2), 155.54 (C6), 148.79 (C2), 147.72 (C4), 139.98 (C8), 124.23 (C5), 85.67 (C1′), 84.26 (C4′), 79.13 (C≡CH), 76.15 (C≡CH), 73.49 (C2′), 71.46 (C3′), 61.33 (C5′), 35.53 (N-CH_2_ propargyl), 27.14 (CH_2_ propionyl), 26.88 (CH_2_ propionyl), 9.40 (CH_3_ propionyl), 9.16 (CH_3_ propionyl). HRMS (ESI) calculated [M+H]^+^ for C_19_H_22_N_4_O_7_: 419.1561, found: 419.1561.

#### 4.2.5. N^1^-Propargyl-5′-O-Methanesulfonyl-2′,3′-di-O-Propionyl Inosine (**5**)

To a cooled (0 °C) solution of compound **4** (0.95 g, 2.3 mmol) in DCM (10 mL) triethylamine (0.41 mL, 3 mmol) and methanesulfonyl chloride (0.23 mL, 3 mmol) were added while stirring. After 12 h of stirring at 25 °C the reaction mixture was diluted with DCM to 100 mL and washed successively with saturated aqueous NaHCO_3_ (50 mL) and water (50 mL). The organic layer was dried over anhydrous Na_2_SO_4_ and concentrated in vacuum. The residue was dissolved in 3 mL of DCM and precipitated with hexane (60 mL). The title compound was purified by column chromatography on silica gel using gradient elution from DCM/hexane 1:1 to DCM/hexane/EtOH 5:5:1 yielding 0.81 g (72%) of mesylate **6** as sticky white foam. R_f_ (B): 0.35. ^1^H-NMR (300.1 MHz, DMSO-*d*_6_): δ = 8.54 (s, 1H, H-2), 8.37 (s, 1H, H-8), 6.23 (d, 1H, *J*_1′,2′_ = 5.7 Hz, H1′), 5.92 (dd, 1H, *J*_2′,3′_ = 5.9 Hz, H2′), 5.61 (dd, 1H, *J*_3′,4′_ = 4.4 Hz, H3′), 4.86 (d, 2H, *J_N_*_-CH2, ≡CH_ = 2.5 Hz, N-CH_2_), 4.61–4.44 (m, 3H, H4′, H5′a, H5′b), 3.41 (t, 1H, ≡CH), 3.19 (s, 3H, CH_3_ mesyl), 2.47–2.38 (m, 2H, CH_2_ propionyl), 2.37–2.28 (m, 2H, CH_2_ propionyl), 1.08 (t, 3H, *J*_CH2, -CH3_ = 7.5 Hz, CH_3_ propionyl), 0.99 (t, 3H, *J*_CH2, -CH3_ = 7.5 Hz, CH_3_ propionyl). ^13^C-NMR (75.5 MHz, DMSO-*d*_6_): 173.09 (O-C=O), 172.88 (O-C=O), 155.54 (C6), 148.80 (C2), 147.64 (C4), 140.39 (C8), 124.42 (C5), 86.09 (C1′), 80.02 (C4′), 79.10 (C≡CH), 76.17 (C≡CH), 72.62 (C2′), 70.13 (C3′), 68.98 (C5′), 37.30 (CH_3_ mesyl), 35.56 (N-CH_2_ propargyl), 27.06 (CH_2_ propionyl), 26.87 (CH_2_ propionyl), 9.31 (CH_3_ propionyl), 9.17 (CH_3_ propionyl). HRMS (ESI) calculated [M+H]^+^ for C_20_H_24_N_4_O_9_S: 497.1337, found: 497.1336.

#### 4.2.6. 3′,5′-di-O-Tert Butyldimethylsilylthymidine, 2′,3′-di-O-Propionylinosine (N3-C5′) Dimer (**6**)

To a stirred solution of compound **5** (0.64 g, 1.3 mmol) and 3′,5′-di-O-t-butyldimethylsilyl thymidine (1.8 g, 3.8 mmol) in acetonitrile (5 mL) 1,8-diazabicyclo[5.4.0]undec-7-ene (0.6 mL 4 mmol) was added. The reaction mixture was left for 48 h at room temperature. Then, the solution was diluted with DCM (150 mL) and washed with water (100 mL). The organic layer was dried over anhydrous Na_2_SO_4_ and concentrated in vacuum. The residue was purified by column chromatography on silica gel using gradient elution from DCM/hexane 1:1 to DCM/hexane/EtOH 50:50:1 yielding 0.46 g (41%) of compound **6**. R_f_ (B): 0.5. ^1^H-NMR (300.1 MHz, DMSO-*d*_6_): δ = 8.52 (s, 1H, H-2 inosine), 8.38 (s, 1H, H-8 inosine), 7.52 (s, 1H, H-6 thymidine), 6.20–6.12 (m, 2H, H1′ inosine, thymidine), 5.94 (dd, 1H, *J*_1′,2′_ = 5.7 Hz, *J*_2′,3′_ = 5.4 Hz, H2′ inosine), 5.57 (dd, 1H, *J*_3′,4′_ = 4.7 Hz, H3′ inosine), 4.85 (d, 2H, *J_N_*_-CH2, ≡CH_ = 2.4 Hz, N-CH_2_ inosine), 4.43–4.28 (m, 3H, H4′, H5′a, H5′b inosine), 4.21–4.08 (m, 1H, H3′ thymidine), 3.85–3.67 (m, 3H, H4′, H5′a, H5′b thymidine), 3.41 (t, 1H, ≡CH inosine), 2.37–2.26 (m, 4H, CH_2_ propionyl), 2.23–2.06 (m, 2H, H2′a, H2′b thymidine), 1.84 (s, 3H, CH_3_ thymidine), 1.04–0.93 (m, 6H, CH_3_ propionyl), 0.88 (s, 18H, CH_3_ tBu), 0.09 (s, 12H, Si-CH_3_). ^13^C-NMR (75.5 MHz, DMSO-*d*_6_): 172.90 (O-C=O), 163.22 (C4 thymidine), 155.54 (C6 inosine), 150.98 (C2 thymidine), 148.60 (C2 inosine), 147.49 (C4 inosine), 140.88 (C8 inosine), 135.01 (C6 thymidine), 124.64 (C5 inosine), 109.13 (C5 thymidine), 87.40 (C1′ thymidine), 86.61 (C1′ inosine), 85.40 (C4′ thymidine), 79.10 (C≡CH), 78.96 (C3′ inosine), 76.18 (C≡CH), 72.77 (C2′ inosine), 72.37 (C3′ thymidine), 72.28 (C4′ inosine), 63.11 (C5′ thymidine), 42.20 (C5′ inosine), 39.91 (C2′, thymidine overlapped with DMSO-d6 signal), 35.51 (N-CH_2_ propargyl), 26.94 (CH_2_ propionyl), 26.87 (CH_2_ propionyl), 26.21 (CH_3_ tBu), 26.11 (CH_3_ tBu), 18.46 (C tBu), 18.14 (CH_3_ tBu), 13.33 (CH_3_ thymidine), 9.23 (CH_3_ propionyl), 9.17 (CH_3_ propionyl), −4.32 (Si-CH_3_), −4.48 (Si-CH_3_), −5.01 (Si-CH_3_). HRMS (ESI) calculated [M+H]^+^ for C_41_H_62_N_6_O_11_Si_2_: 871.4088, found: 871.4090.

#### 4.2.7. Thymidine, 2′,3′-di-O-Propionylinosine (N3-C5′) Dimer (**7**)

To a solution of compound **6** (0.42 g, 0.48 mmol) in THF (2 mL) triethylamine (0.65 mL) and triethylamine trihydrofluoride (0.65 mL) were added. After 4 h the reaction mixture was diluted with DCM (100 mL) and washed with 10% aqueous sodium chloride (50 mL). The organic layer was dried over anhydrous Na_2_SO_4_ and concentrated under reduced pressure. The residue was purified by column chromatography on silica gel using gradient elution from DCM/hexane/EtOH 10:10:1 to DCM/EtOH 9:1 yielding 0.28 g (91%) of compound **7**. R_f_ (A): 0.8. ^1^H-NMR (300.1 MHz, DMSO-*d*_6_): δ = 8.51 (s, 1H, H-2 inosine), 8.37 (s, 1H, H-8 inosine), 7.81 (s, 1H, H-6 Thy), 6.20–6.12 (m, 2H, H1′ inosine, thymidine), 5.94 (dd, 1H, *J*_1′,2′_ = 5.6 Hz, *J*_2′,3′_ = 5.4 Hz, H2′ inosine), 5.57 (dd, 1H, *J*_3′,4′_ = 4.8 Hz, H3′ inosine), 5.23 (d, 1H, *J*_3′-OH_ = 4.3 Hz, 3′-OH thymidine), 5.04 (t, 1H, *J*_5′a-OH_ = *J*_5′b-OH_ = 5.2 Hz, 5′-OH thymidine), 4.85 (d, 2H, *J_N_*_-CH2, ≡CH_ = 2.4 Hz, N-CH_2_), 4.43–4.09 (m, 4H, H4′, H5′a, H5′b inosine, H3′ thymidine), 3.79 (dd, 1H, *J*_4′,3′_ = *J*_4′,5′a_ = *J*_4′,5′b_ = 3.5 Hz, H4′ thymidine), 3.67–3.52 (m, 2H, H5′a, H5′b thymidine), 3.41 (t, 1H, ≡CH), 2.37–2.27 (m, 4H, CH_2_ propionyl), 2.16–2.05 (m, 2H, H2′a, H2′b thymidine), 1.83 (s, 3H, CH_3_ thymidine), 1.06–0.93 (m, 6H, CH_3_ propionyl). ^13^C-NMR (75.5 MHz, DMSO-*d*_6_): 172.93 (O-C=O), 172.89 (O-C=O), 163.30 (C4 thymidine), 155.56 (C6 inosine), 151.05 (C2 thymidine), 148.59 (C2 inosine), 147.49 (C4 inosine), 140.87 (C8 inosine), 135.56 (C6 thymidine), 124.63 (C5 inosine), 108.89 (C5 thymidine), 87.94 (C1′ thymidine), 86.61 (C1′ inosine), 85.45 (C4′ thymidine), 79.11 (C≡CH), 79.03 (C3′ inosine), 76.18 (C≡CH), 72.78 (C2′ inosine), 72.25 (C4′ inosine), 70.66 (C3′ thymidine), 61.62 (C5′ thymidine), 42.14 (C5′ inosine), 40.16 (C2′ thymidine, overlapped by the DMSO-d6 signal), 35.52 (N-CH_2_ propargyl), 26.97 (CH_2_ propionyl), 26.87 (CH_2_ propionyl), 13.33 (CH_3_ thymidine), 9.28 (CH_3_ propionyl), 9.18 (CH_3_ propionyl). HRMS (ESI) calculated [M+H]^+^ for C_29_H_34_N_6_O_11_: 643.2358, found: 643.2358.

#### 4.2.8. 5′-O-Dimethoxytrityl Thymidine, 2′,3′-di-O-Propionylinosine (N3-C5′) Dimer (**8**)

Compound **7** (0.27 g, 0.42 mmol) was treated with 4,4′-dimethoxytrityl chloride (0.15 g, 0.45 mmol) in pyridine (5 mL) for 24 h at room temperature. Pyridine was removed in vacuum. The residue was dissolved in DCM (100 mL) and washed successively with saturated aqueous NaHCO_3_ (50 mL) and water (50 mL). The organic layer was dried over anhydrous Na_2_SO_4_ and concentrated under reduced pressure. The residue was purified by column chromatography on silica gel using gradient elution from DCM/hexane 1:1 to DCM/hexane/EtOH 10:10:1 yielding 0.31 g (78%) of compound **8**. R_f_ (B): 0.65. ^1^H-NMR (300.1 MHz, DMSO-*d*_6_): δ = 8.52 (s, 1H, H-2 inosine), 8.37 (s, 1H, H-8 inosine), 7.58 (s, 1H, H-6 thymidine), 7.62–7.18 and 6.91–6.88 (m, 13H aromatic), 6.22 (dd, 1H *J*_1′,2a′_ = 6.5 Hz, *J*_1′,2b′_ = 6.8 Hz, H1′ thymidine), 6.16 (d, 1H, *J*_1′,2′_ = 5.1 Hz, H1′ inosine), 5.94 (dd, 1H, *J*_2′,3′_ = 5.8 Hz, H2′ inosine), 5.59 (dd, 1H, *J*_3′,4′_ = 4.4 Hz, H3′ inosine), 5.34 (d, 1H, *J*_3′-OH_ = 4.6 Hz, 3′-OH thymidine), 4.84 (d, 2H, *J_N_*_-CH2, ≡CH_ = 2.4 Hz, N-CH_2_), 4.42–4.28 (m, 3H, H4′, H5′a inosine, H3′ thymidine), 4.23–4.12 (m, 1H, H5′b inosine), 3.92 (ddd, 1H, *J*_4′,3′_ = 3.5 Hz, *J*_4′,5′a_ = 4.4 Hz, *J*_4′,5′b_ = 2.9 Hz, H4′ thymidine), 3.73 (s, 6H, OCH_3_), 3.40 (t, 1H, ≡CH), 3.28–3.15 (m, 2H, H5′a, H5′b thymidine), 2.38–2.16 (m, 6H, H2′a, H2′b thymidine, CH_2_ propionyl), 1.50 (s, 3H, CH_3_ thymidine), 1.07–0.93 (m, 6H, CH_3_ propionyl). ^13^C-NMR (75.5 MHz, DMSO-*d*_6_): 172.90 (O-C=O), 163.22 (C4 thymidine), 158.64 (C4 PhOMe), 155.55 (C6 inosine), 151.00 (C2 thymidine), 148.60 (C2 inosine), 147.49 (C4 inosine), 145.15 (C1 Ph), 140.83 (C8 inosine), 135.91 (C1 Ph-OMe), 135.73 (C1 Ph-OMe), 135.15 (C6 thymidine), 130.20 (C2,6 Ph-OMe), 127.26 (C4 Ph), 128.36 (C3 Ph), 128,15 (C5 Ph), 124.62 (C5 inosine), 113.73 (C3,5 Ph-OMe), 109.19 (C5 thymidine), 86.65 (C1′ inosine), 86.35 (C1′ thymidine), 86.14 (C4′ thymidine), 85.36 (O-C DMTr), 79.10 (C≡CH), 78.85 (C3′ inosine), 76.16 (C≡CH), 72.87 (C2′ inosine), 72.31 (C4′ inosine), 70.82 (C3′ thymidine), 64.15 (C5′ thymidine), 55.52 (O-CH_3_ DMTr), 42.31 (C5′ inosine), 40.16 (C2′ thymidine overlapped with the DMSO-d6 signal), 35.52 (N-CH_2_ propargyl), 26.95 (CH_2_ propionyl), 26.87 (CH_2_ propionyl), 12.79 (CH_3_ thymidine), 9.24 (CH_3_ propionyl), 9.17 (CH_3_ propionyl). HRMS (ESI) calculated [M+H]^+^ for C_50_H_52_N_6_O_13_: 945.3665, found: 945.3655.

#### 4.2.9. 5′-O-Dimethoxytritylthymidine-3′-O-(2-Cyanoethyl-N,N-Diisopropyl)phosphoramidite, 2′,3′-di-O-Propionylinosine (N3-C5′) Dimer (**9**)

Compound **8** (0.3 g, 0.32 mmol), pyridine (25 μL) and tetrazole (25 mg, 0.35 mmol) were dissolved in 3 mL of anhydrous MeCN, and 3–5 beads of 4 Å molecular sieves (4–8 mesh) were added to the solution. The mixture was flushed with argon and left for 30 min. Then, 2-cyanoethyl N,N,N′,N′-tetraisopropylphosphorodiamidite (105 μL, 0.35 mmol) was added under intensive stirring to the solution. The reaction was allowed to proceed for 30 min at room temperature. Then, the solution was diluted with ethyl acetate (50 mL) and washed twice with cold saturated aqueous NaHCO_3_ (50 mL) and cold water (50 mL). The organic layer was dried over anhydrous Na_2_SO_4_, and the solvent was removed in vacuum. The residue was purified by column chromatography on silica gel using gradient elution with an ethyl acetate/hexane/triethylamine mixture from 50:50:1 to 70:30:1 yielding 0.3 g (83%) of compound **9**. R_f_ (C): 0.55. ^1^H-NMR (300.1 MHz, DMSO-*d*_6_): δ = 8.52 (s, 1H, H-2 inosine), 8.39 (s, 1H, H-8 inosine), 7.62, 7.20 (s, 1H, H-6 thymidine diaster. I+II), 7.45–7.19 and 6.95–6.83 (m, 13H, aromatic), 6.24, 6.22 (t, 1H *J*_1′,2a′_ = *J*_1′,2b′_ = 6.5 Hz, H1′ thymidine diaster. I+II), 6.16 (d, 1H, *J*_1′,2′_ = 5.1 Hz, H1′ inosine), 5.94 (dd, 1H, *J*_2′,3′_ = 5.8 Hz, H2′ inosine), 5.59, 5.57 (dd, 1H, *J*_3′,4′_ = 8.0 Hz, H3′ inosine), 4.85 (d, 2H, *J_N_*_-CH2, ≡CH_ = 2.4 Hz, N-CH_2_), 4.62–4.49 (m, 1H, H3′ thymidine diaster. I+II), 4.43–4.30 (m, 2H, H4′, H5′a inosine), 4.23–3.98 (m, 2H, H5′b inosine, H4′ thymidine), 3.73 (s, 6H, OCH_3_), 3.66–3.45 (m, 3H, H5′a, H5′b, CH(CH_3_)_2_ thymidine), 3.41 (t, 1H, ≡CH), 3.29–3.22 (m, 1H, CH(CH_3_)_2_), 2.77, 2.64 (t 2H, *J*_CH2-C≡N, O-CH2_ = 5.9 Hz, CH_2_CN), 2.44–2.25 (m, 6H, H2′a, H2′b thymidine, CH_2_ propionyl), 1.54 (s, 3H, CH_3_ thymidine diaster. I+II), 1.18–0.93 (m, 18H, CH_3_ propionyl, isopropyl). ^13^C-NMR (75.5 MHz, DMSO-*d*_6_): 172.90 (O-C=O), 163.20 (C4 thymidine), 158.69 (C4 Ph-OMe), 155.55 (C6 inosine), 150.95 (C2 thymidine), 148.61 (C2 inosine), 147.50 (C4 inosine), 145.03 (C1 Ph), 140.84 (C8 inosine), 135.77 (C1 Ph-OMe), 135.63, 135.60 (C6 thymidine diaster. I, II), 135.06 (C1 Ph-OMe), 130.22 (C2,6 Ph-OMe), 128.35 (C4 Ph), 128,17 (C3,5 Ph), 127.32 (C4 Ph), 124.63 (C5 inosine), 119.39, 119.19 (CN diaster. I, II), 113.70 (C3,5 Ph-OMe), 109.39, 109.31 (C5 thymidine diaster. I, II), 86.61 (C1′ inosine), 86.50, 86.44 (C1′ thymidine diaster. I, II), 85.38, 85.31 (C4′ thymidine diaster. I, II, O-C DMTr), 85.08, 85.04 (C3′ thymidine diaster. I, II), 79.11 (C≡CH), 78.92, 78.88 (C3′ diaster. I, II, inosine), 76.17 (C≡CH), 72.83 (C2′ inosine), 72.32 (C4′ inosine), 63.66, 63.50 (C5′ thymidine diaster. I, II), 58.92, 58.67 (-CH_2_- cyanoethyl diaster. I, II), 55.53 (O-CH_3_ DMTr), 42.31 (C5′ inosine), 39.07 (C2′ thymidine overlapped by the DMSO-d6 signal), 35.52 (N-CH_2_), 26.96 (CH_2_ propionyl), 26.87 (CH_2_ Propionyl), 24.85, 24.74, 24.70, 24.64 (CH_3_ isopropyl diaster. I, II), 12.81 (CH_3_ thymidine), 9.24, 9.17 (CH_3_ propionyl). ^31^P-NMR (202 MHz, DMSO-*d*_6_): 147.74 (diaster. I) and 147.40 (diaster. II). HRMS (ESI) calculated [M+H]^+^ for C_59_H_69_N_8_O_14_P: 1145.4744, found: 1145.4753.

### 4.3. Oligonucleotide Synthesis and Modifications

DNA oligomers were synthesized using an ABI 3400 DNA/RNA synthesizer (Applied Biosystems, Foster City, CA, USA in DMTr-on mode. The coupling time of the modified TID phosphoramidite was 60 s. Partial deprotection of the oligonucleotides was carried out with concentrated ammonia for 6 h at 55 °C. DMTr-protected oligonucleotides were purified by reverse-phase HPLC (Hypersil ODS (Thermo Fisher Scientific, Waltham, MA, USA), 5 μm, 4.6 × 250 mm; 10–50% MeCN in 50 mM TEAA for 30 min; 1 mL/min). After removal of the 5′ DMTr group, the oligomers were repeatedly purified by reversed-phase HPLC (0–25% MeCN in 50 mM TEAA for 30 min).

Click conjugation of the 12-mer TID-oligomer with small molecule azides was performed with 20 nmol of oligomer and 1 mg of the respective azide in 10 mM TEAA in 80% aqueous DMF (90 μL). The reaction mixture was supplemented with 100 mM sodium ascorbate (5 μL) and flushed with argon for 30 s. Then, Cu(II)-TBTA complex (5 μL) was added, and the reaction mixture was flushed with argon. After 24 h, the oligonucleotide was precipitated with 2% LiClO_4_ in acetone (1 mL), washed with acetone, and dried. The pellet was dissolved in μQ water (0.5 mL), and oligonucleotide conjugate was purified by reversed-phase HPLC (0–100% MeCN in 50 mM TEAA for 60 min).

Oligonucleotide azide derivatives were prepared by reacting excess N3-PEG6-NHS ester with 5′-aminomodified oligonucleotide in 0.1 M sodium carbonate buffer (pH 9) at 4 °C for 18 h. Oligonucleotide azides were purified by reverse phase HPLC.

Branched oligonucleotide scaffolds were prepared by click conjugation of TID-oligomers with oligonucleotide azides at a 50 nmol scale. The oligonucleotide ratio was 1:1. The oligonucleotide mixture was dried in a vacuum concentrator and diluted in 20 μL of 10 mM TEAA in 50% aqueous DMF. The mixture was supplemented with 100 mM sodium ascorbate (10 μL) and flushed with argon for 30 s. Then, the Cu(II)-TBTA complex (10 μL) was added, and the reaction mixture was flushed with argon. After 24 h, oligonucleotide scaffold was precipitated and purified by reverse phase HPLC as described for small molecule conjugates.

The primer for PEX was prepared using N3-propargyl thymidine phosphoramidite [17] at the last coupling step. After purification by the reversed-phase HPLC, the oligomer was conjugated to Cy5-azide by a click reaction (see above) using an equimolar ratio of primer to Cy5-azide. The reaction was carried out in 100 μL of 50% aqueous DMF. Cy5-labeled primer was purified by reverse phase HPLC.

### 4.4. Oxidative Cleavage of TID Oligonucleotide Derivatives

The TID oligonucleotide derivative (12–20 nmol) was treated with 100 mM aqueous NaIO_4_ or Et_3_N-HIO_4_ in a total volume of 50 μL for 10 min. The mixture was subsequently diluted with 20% triethylamine in 50% aqueous acetonitrile (1 mL) and heated at 55 °C. For kinetic studies, aliquots were taken after heating for various periods of time, dried in a vacuum concentrator, and analyzed by electrophoresis in a 20% cross-linked polyacrylamide gel (19:1) under denaturing conditions (7M urea, 1xTBE). Alternatively, heating was performed in 30% aqueous ammonia at 55 °C or in 5% aqueous 1,1,3,3,-tetramethyl guanidine (TMG) at 37 °C. In the latter case, aliquots for PAGE analysis were taken after heating for various periods of time and TMG was removed by precipitation with 2% LiClO_4_ in acetone. Samples were dissolved in loading buffer and analyzed by electrophoresis under denaturing conditions. For HPLC analysis, aliquots were diluted in 50 mM aqueous TEAA. HPLC analysis of the cleavage products was carried out with a HiBar C18 column (250–4, 5 Å), (Merck, Darmstadt, Germany) using a linear gradient of MeCN (0–50%) in 50 mM aqueous TEAA over 45 min. The flow rate was 1 mL/min.

### 4.5. Primer Extension Reaction

The reaction mixture (25 μL) contained 4 μM Cy5-labeled primer and 4 μM template strand. The dNTP concentration was 200 μM (for each). The final concentration of DNA polymerase was 0.1 U/μL. The template strand and Cy5-labeled primer were annealed in ThermoPol^®^ buffer (New England Biolabs, Ipswich, MA, USA) from 95 to 5 °C. Klenow DNA polymerase and dNTPs were then added. The PEX reaction was carried out for 1 h at 37 °C. The reaction was stopped by precipitation with 2% LiClO_4_ in acetone. Strand separation from PEX reactions was performed in a 20% polyacrylamide (19:1) gel containing 1xTBE and 7 M urea at 56 °C using water jacketed electrophoretic unit (Thermo Fisher Scientific, Waltham, MA, USA). Fluorescent bands were visualized with a GenoSens 2250 (Clinx Science Instruments, Shanghai, China).

### 4.6. Anchoring of TID-Dodecanucleotide to HybCPG Support and Enzymatic Labeling with Cy5-dUTP

HybCPG-3-amine support (Prime Synthesis Inc., Aston, PA, USA) (50 mg) was treated with azide-PEG6-NHS linker (20 μL) and DIPEA (30 μL) in 1.5 mL of DCM for 3 h at 25 °C. The support was separated by filtration, washed with acetone and treated with acetic anhydride in pyridine (1:2, 1 mL) for 5 min. Derivatized CPG was washed with acetone and dried on a glass filter. TID-modified dodecamer (10 nmol) was dissolved in 50 μL of 10 mM TEAA in 50% aqueous DMF and added to 5 mg of derivatized GPG. The suspension was supplemented with 100 mM sodium ascorbate (5 μL) and flushed with argon for 30 s. Then Cu(II)-TBTA complex (5 μL) was added, and the reaction mixture was flushed with argon. After 24 h, the support was filtered and washed with milli-Q water at room temperature and briefly (1 min) at 80 °C. Cleavage of the oligonucleotide from HybCPG support was carried out by the oxidative cleavage as described in Section 4.4. Quantification of the recovered oligomer was performed using Implen NanoPhotometer N60 UV-Vis Spectrophotometer (Implen GMBH, München, Germany).

Dodecamer-functionalized HybCPG (3 mg) was suspended in 1× TdT buffer (50 μL). Cy5-dUTP (1 mM, 1 μL) and TdT (30 U) were added to the solution, and the mixture was heated at 37 °C for 2 h. Underivatized HybCPG was used in the TdT labeling reaction as a negative control. After labeling, the support was washed three times with milli-Q water. Cy5 fluorescence was analyzed using a GenoSens 2250 instrument (Clinx Science Instruments, Shanghai, China).

## Figures and Tables

**Table 1:** Thermal stabilities of DNA duplexes CXAGATACCGAT/ATCGGTATCTYG in 1 M NaCl and 10 mM sodium phosphate (pH 7.5).

Y	*T*_m_, °C, (X = T)	Δ*T*_m_ *	*T*_m_, °C, (X = TID)	Δ*T*_m_ *
A	48.7 ± 0.9		45.2 ± 0.6	3.5
G	43.9 ± 0.6	4.8	44.7 ± 0.3	4.1
C	43.5 ± 1.4	5.3	43.6 ± 0.4	5.2
T	43.4 ± 0.4	5.3	44.0 ± 0.4	4.8
	average	5.1	average	4.4

* Referenced to the perfect DNA duplex (XY = TA).

## Data Availability

The original contributions presented in the study are included in the article and the Supplementary Materials. Further inquiries can be directed to the corresponding author.

## Supplementary Materials

The following supporting information can be downloaded at: https://www.mdpi.com/article/10.3390/molecules30183769/s1, Figure S1: MALDI MS analysis of TID-modified dodecamer 5′-C-TID-AGATACCGAT; Figure S2: Small molecule azide derivatives; Figure S3: Anchoring of TID-modified dodecamer to a solid support, recovery of unmodified dodecamer, and enzymatic labeling of support-bound dodecamer using TdT and Cy5-dUTP; Figure S4: Analysis of unbound and cleaved dodecanucleotides by denaturing PAGE; Figure S5: MALDI analysis of a sample of the anthracene-modified oligomer after oxidation and heating in concentrated aqueous ammonia for 30 min at 55 °C; ^1^H, ^13^C, and ^31^P NMR and HRMS of new compounds.
