# Supplementary material for: Thymidine-Inosine Dimer Building Block for Reversible Modification of Synthetic Oligonucleotides
Source: Molecules. 2025 Sep 17;30(18):3769. doi: 10.3390/molecules30183769 (PMC12472319; doi:10.3390/molecules30183769)

# Thymidine-Inosine Dimer Building Block for Reversible Modification of Synthetic Oligonucleotides

**Natalia A. Kolganova, Irina V. Varizhuk, Andrey A. Stomakhin, Marat M. Khisamov, Pavel N. Solyev, Sergei A. Surzhikov and Edward N. Timofeev ***

W.A. Engelhardt Institute of Molecular Biology, Russian Academy of Sciences, Vavilov St. 32, Moscow 119991, Russia

| | |
|---|---|
| Supplementary Figure S1 | S2 |
| Supplementary Figure S2 | S2 |
| Supplementary Figure S3 | S3 |
| Supplementary Figure S4 | S3 |
| Supplementary Figure S5 | S4 |
| NMR spectra | S5 |
| HRMS spectra | S15 |

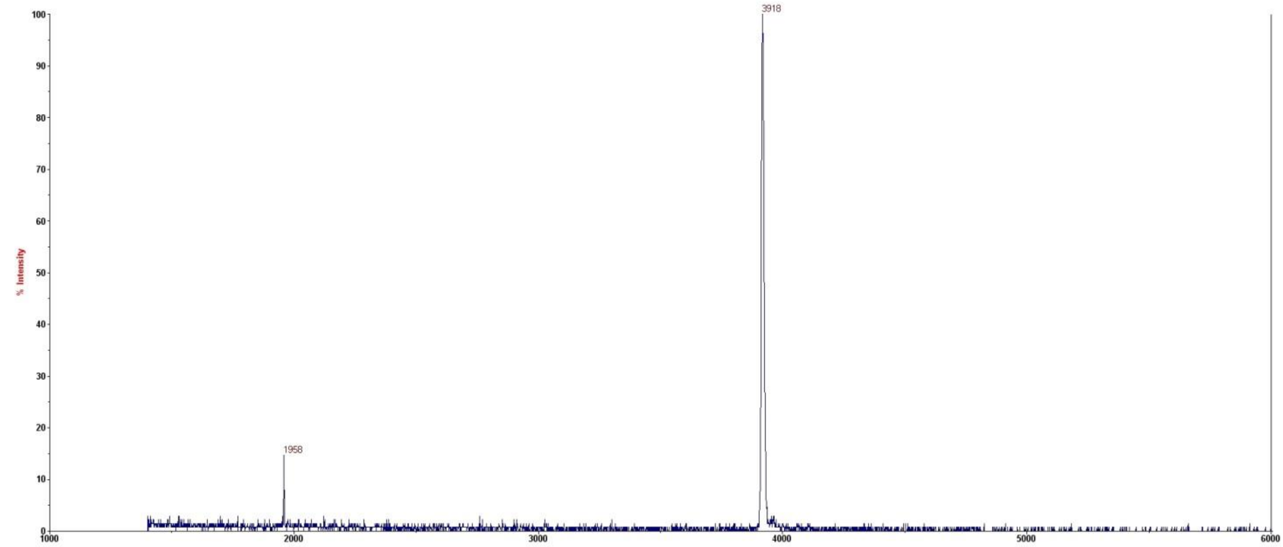


Supplementary Figure S1. MALDI MS analysis of TID-modified dodecamer 5'-C-TID-AGATACCGAT. Calculated/observed $[MH]^+$: 3917/3918 Da.

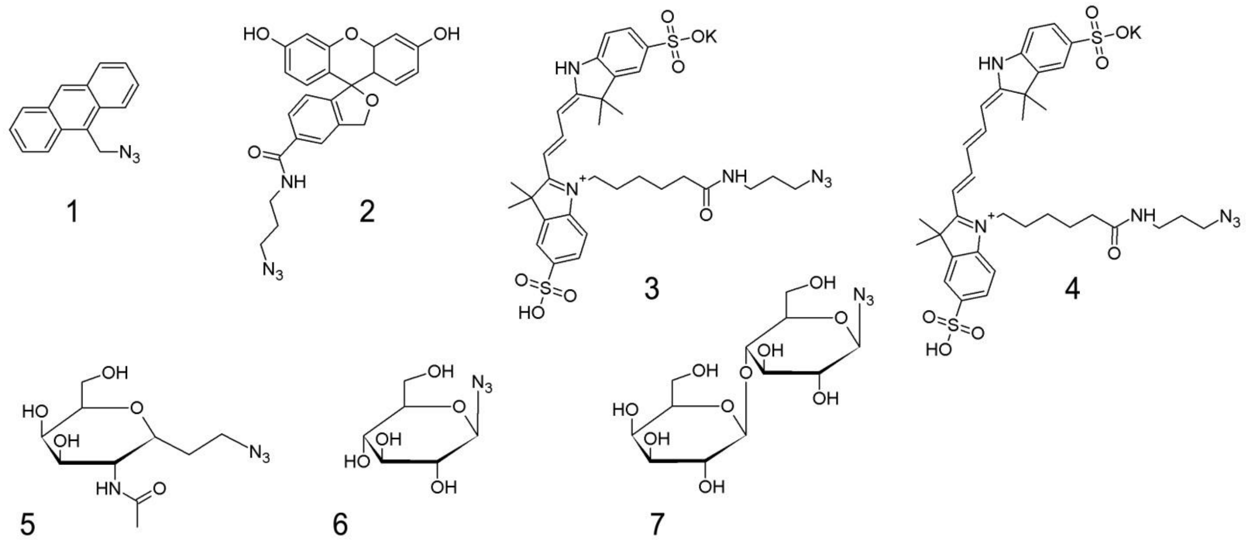


Supplementary Figure S2. Small molecule azide derivatives. Anthracene (1), fluorescein (2), sulfo-Cy3 (3), sulfo-Cy5 (4), GalNAc (5), 1-β-D-glycopyranose (6), and 1-β-D-lactopyranose (7).

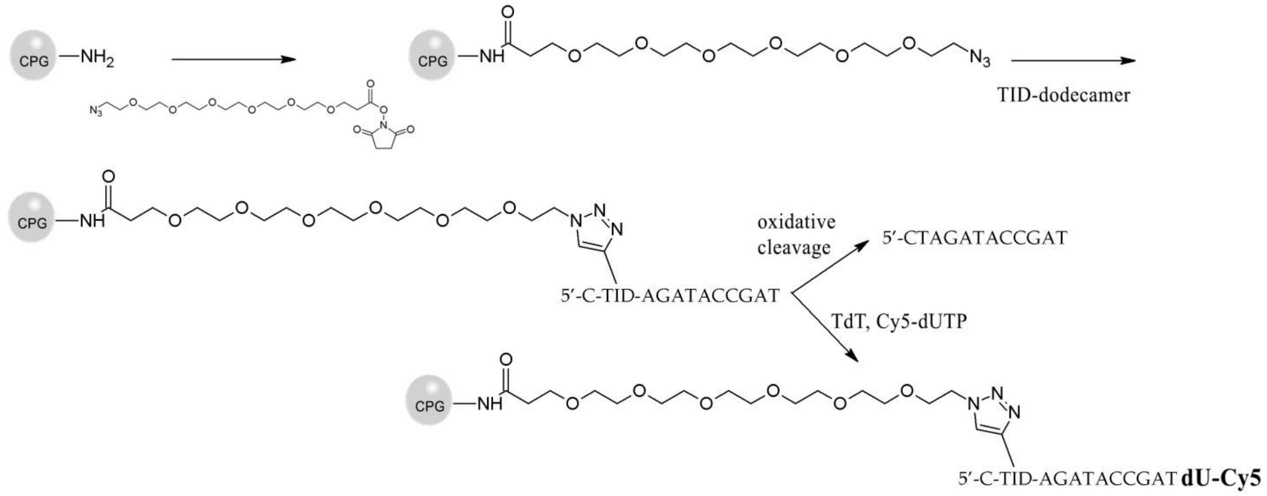


Supplementary Figure S3. Anchoring of TID-modified dodecamer to a solid support, recovery of unmodified dodecamer, and enzymatic labeling of support-bound dodecamer using TdT and Cy5-dUTP.

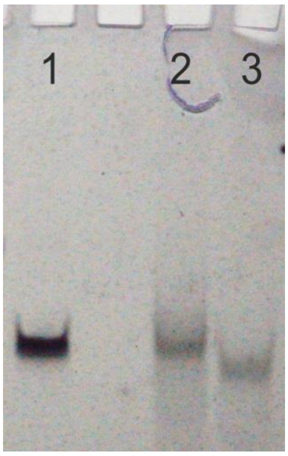


Supplementary Figure S4. Analysis of unbound and cleaved dodecanucleotides by denaturing PAGE. (1) TID-modified dodecamer; (2) Unbound TID-modified dodecamer; (3) Unmodified dodecamer recovered from functionalized CPG support by oxidative cleavage.

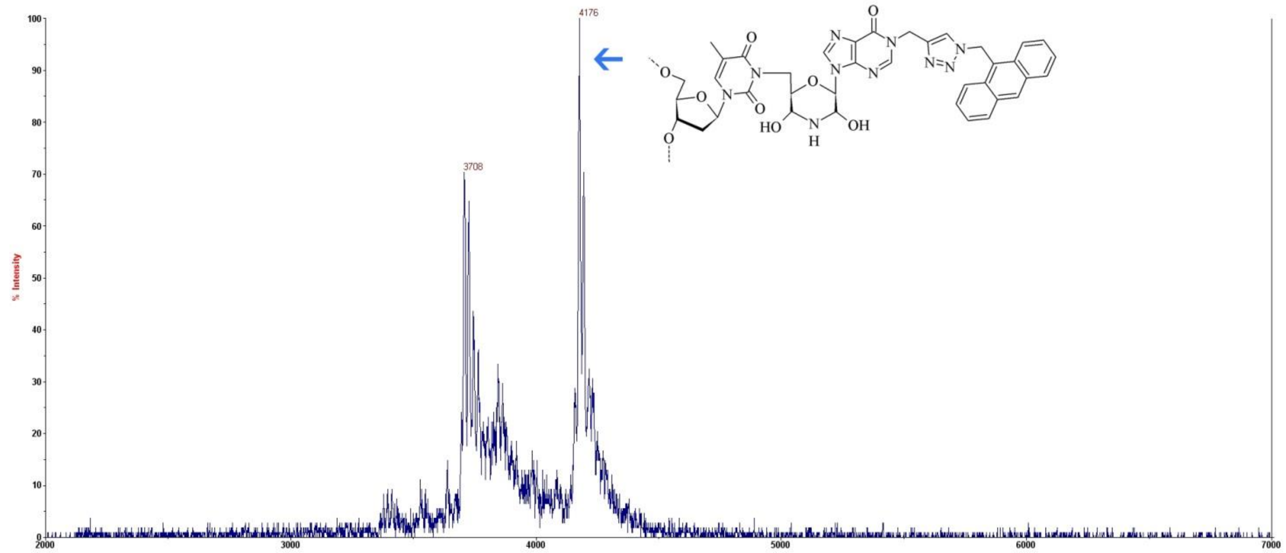


Figure S5. MALDI analysis of a sample of the anthracene-modified oligomer after oxidation and heating in concentrated aqueous ammonia for 30 min at 55 °C. Calculated/observed $[MH]^+$ for the morpholino-type intermediate: 4166/4176 Da.

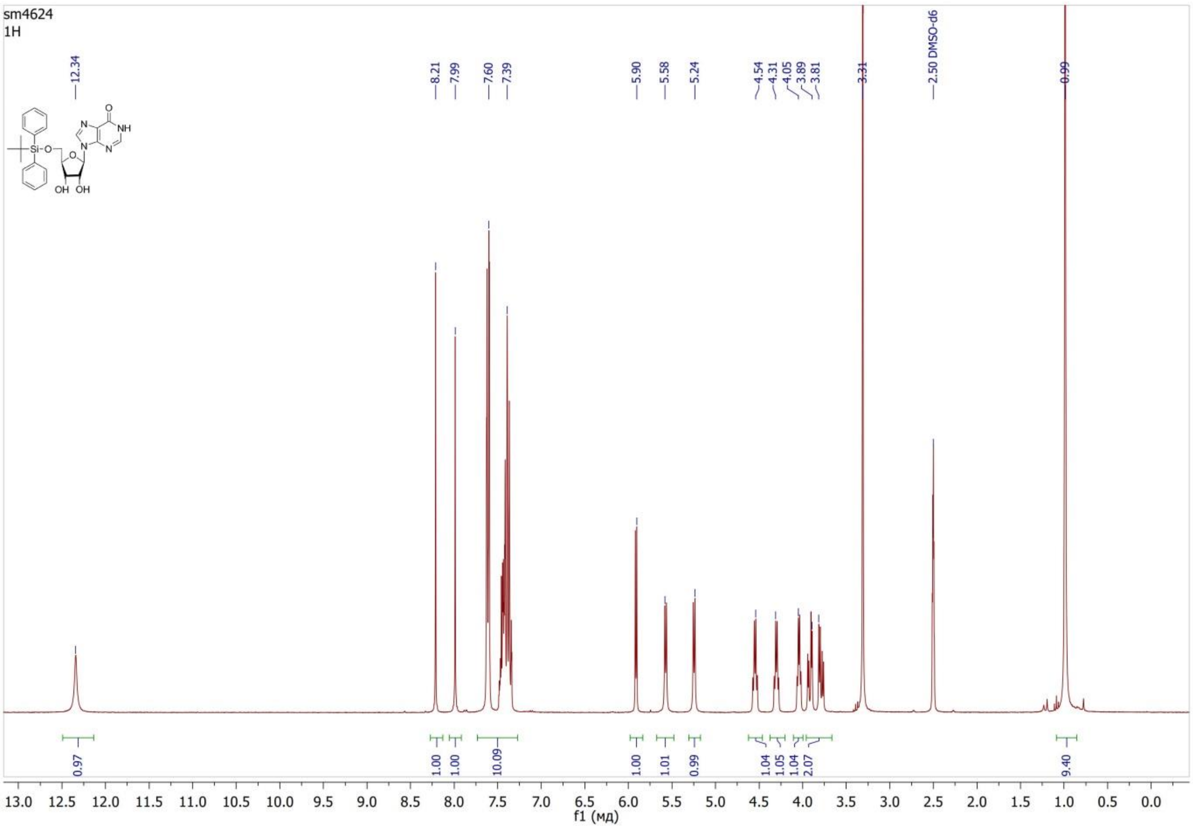
sm4624
1H
12.34
8.21
7.99
7.60
7.39
5.90
5.58
5.24
4.54
4.31
4.05
3.89
3.81
3.31
2.50 DMSO-d6
0.99
0.97
1.00
1.00
10.09
1.00
1.01
0.99
1.04
1.05
1.04
2.07
9.40
13.0 12.5 12.0 11.5 11.0 10.5 10.0 9.5 9.0 8.5 8.0 7.5 7.0 6.5 6.0 5.5 5.0 4.5 4.0 3.5 3.0 2.5 2.0 1.5 1.0 0.5 0.0
f1 (мд)

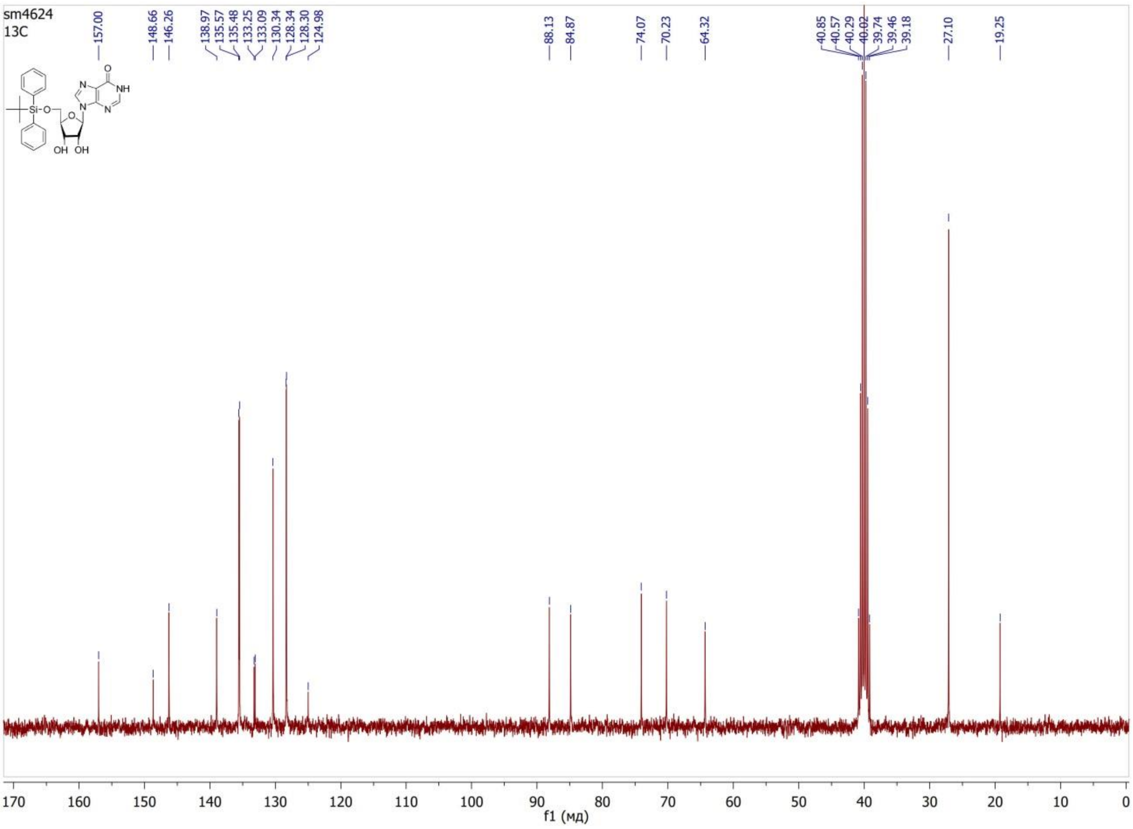
sm4624
13C
157.00
148.66
146.26
138.97
135.57
135.48
133.25
133.09
130.34
128.34
128.30
124.98
88.13
84.87
74.07
70.23
64.32
40.85
40.57
40.29
40.02
39.74
39.46
39.18
27.10
19.25
170 160 150 140 130 120 110 100 90 80 70 60 50 40 30 20 10 0
f1 (мд)

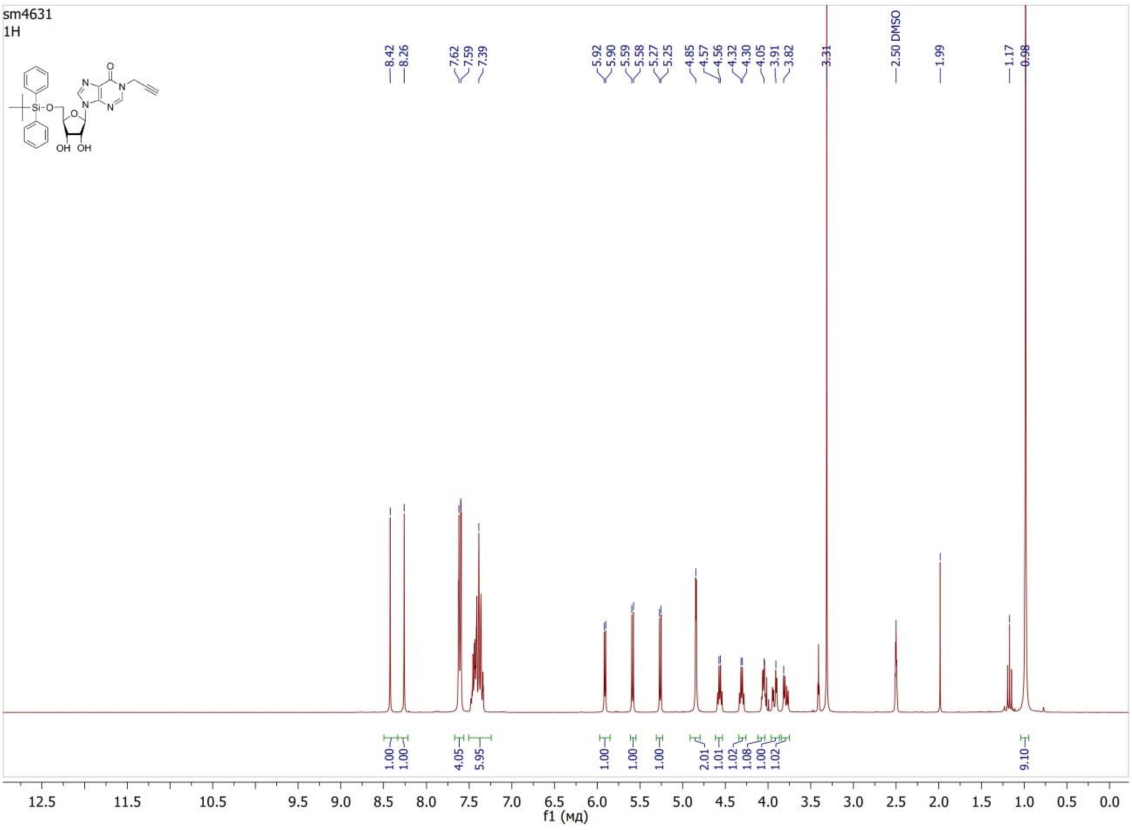
sm4631
1H
8.42
8.26
7.62
7.59
7.39
5.92
5.90
5.59
5.58
5.27
5.25
4.85
4.57
4.56
4.32
4.30
4.05
3.91
3.82
3.31
2.50 DMSO
1.99
1.17
0.98
1.00
1.00
4.05
5.95
1.00
1.00
1.00
2.01
1.01
1.02
1.08
1.00
1.02
9.10
f1 (мд)

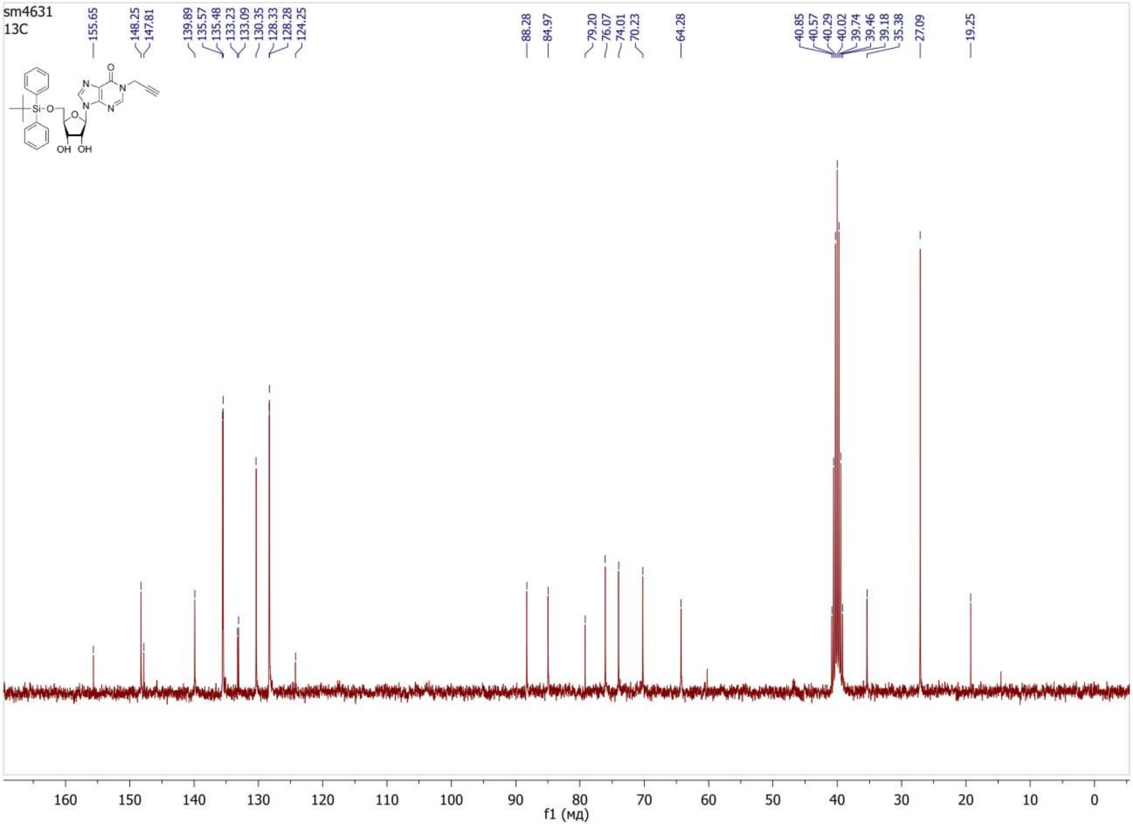
sm4631
13C
155.65
148.25
147.81
139.89
135.57
135.48
133.23
133.09
130.35
128.33
128.28
124.25
88.28
84.97
79.20
76.07
74.01
70.23
64.28
40.85
40.57
40.29
40.02
39.74
39.46
39.18
35.38
27.09
19.25
f1 (мд)

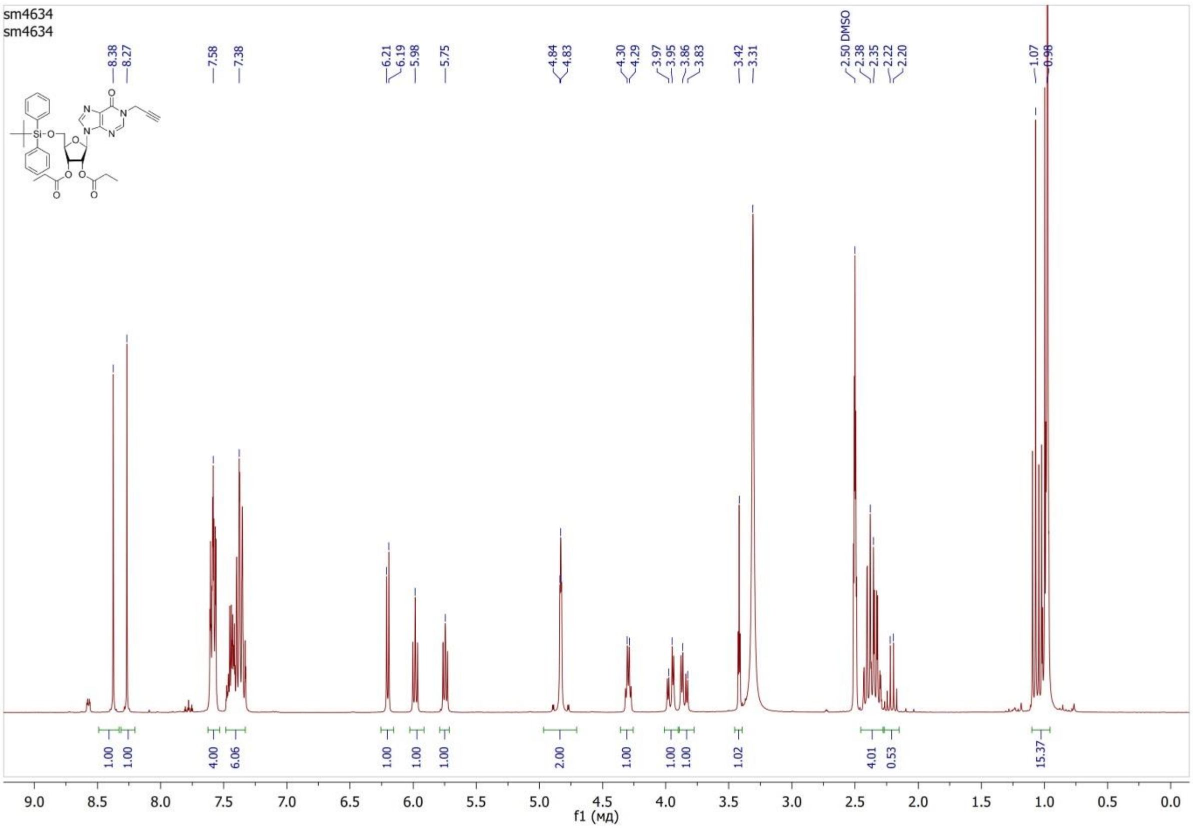
sm4634
sm4634
8.38
8.27
7.58
7.38
6.21
6.19
5.98
5.75
4.84
4.83
4.30
4.29
3.97
3.95
3.86
3.83
3.42
3.31
2.50 DMSO
2.38
2.35
2.22
2.20
1.07
0.98
1.00
1.00
4.00
6.06
1.00
1.00
1.00
2.00
1.00
1.00
1.00
1.02
4.01
0.53
15.37
9.0 8.5 8.0 7.5 7.0 6.5 6.0 5.5 5.0 4.5 4.0 3.5 3.0 2.5 2.0 1.5 1.0 0.5 0.0
f1 (мд)

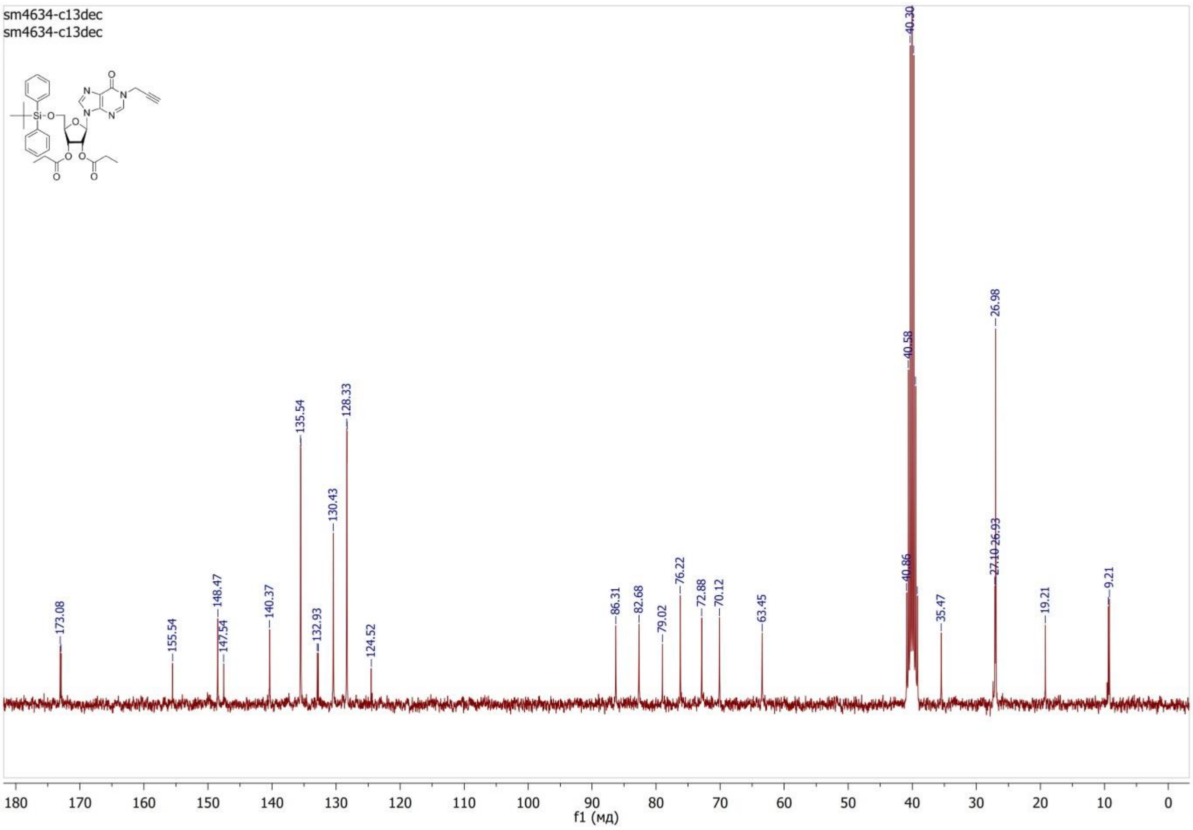
sm4634-c13dec
sm4634-c13dec
173.08
155.54
148.47
147.54
140.37
135.54
132.93
130.43
128.33
124.52
86.31
82.68
79.02
76.22
72.88
70.12
63.45
40.86
40.58
40.30
35.47
27.10 26.93
26.98
19.21
9.21
180 170 160 150 140 130 120 110 100 90 80 70 60 50 40 30 20 10 0
f1 (мд)

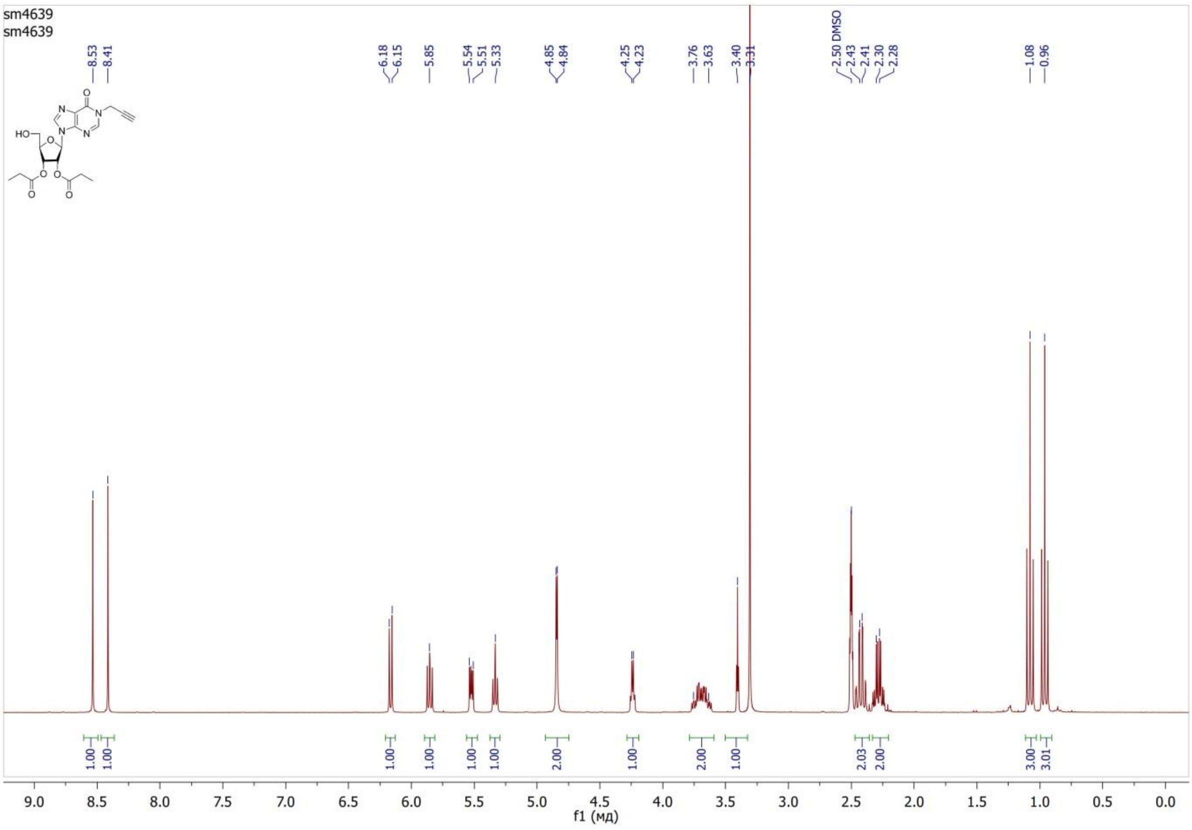

sm4639
sm4639
8.53
8.41
6.18
6.15
5.85
5.54
5.51
5.33
4.85
4.84
4.25
4.23
3.76
3.63
3.40
3.31
2.50 DMSO
2.43
2.41
2.30
2.28
1.08
0.96
1.00
1.00
1.00
1.00
1.00
1.00
2.00
1.00
2.00
1.00
2.03
2.00
3.00
3.01
9.0 8.5 8.0 7.5 7.0 6.5 6.0 5.5 5.0 4.5 4.0 3.5 3.0 2.5 2.0 1.5 1.0 0.5 0.0
f1 (мд)


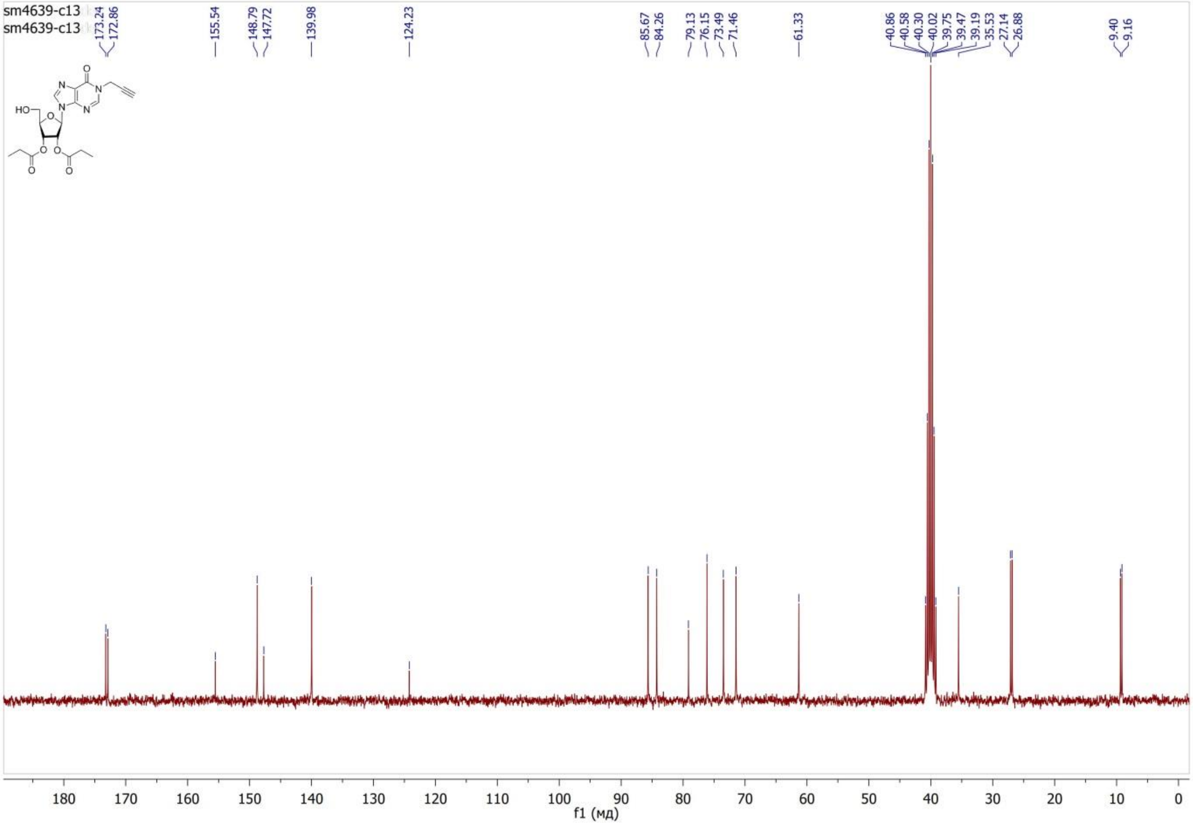

sm4639-c13
sm4639-c13
173.24
172.86
155.54
148.79
147.72
139.98
124.23
85.67
84.26
79.13
76.15
73.49
71.46
61.33
40.86
40.58
40.30
40.02
39.75
39.47
39.19
35.53
27.14
26.88
9.40
9.16
180 170 160 150 140 130 120 110 100 90 80 70 60 50 40 30 20 10 0
f1 (мд)

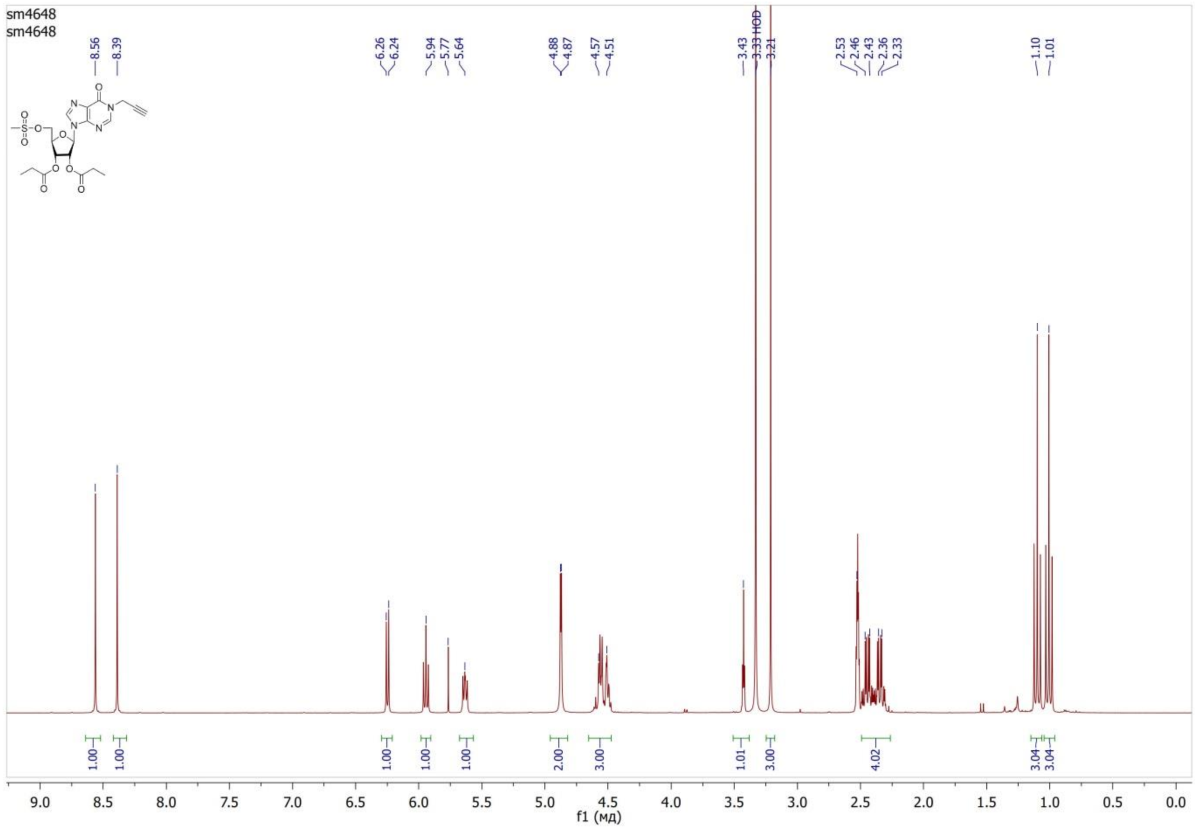
sm4648
sm4648
f1 (мд)

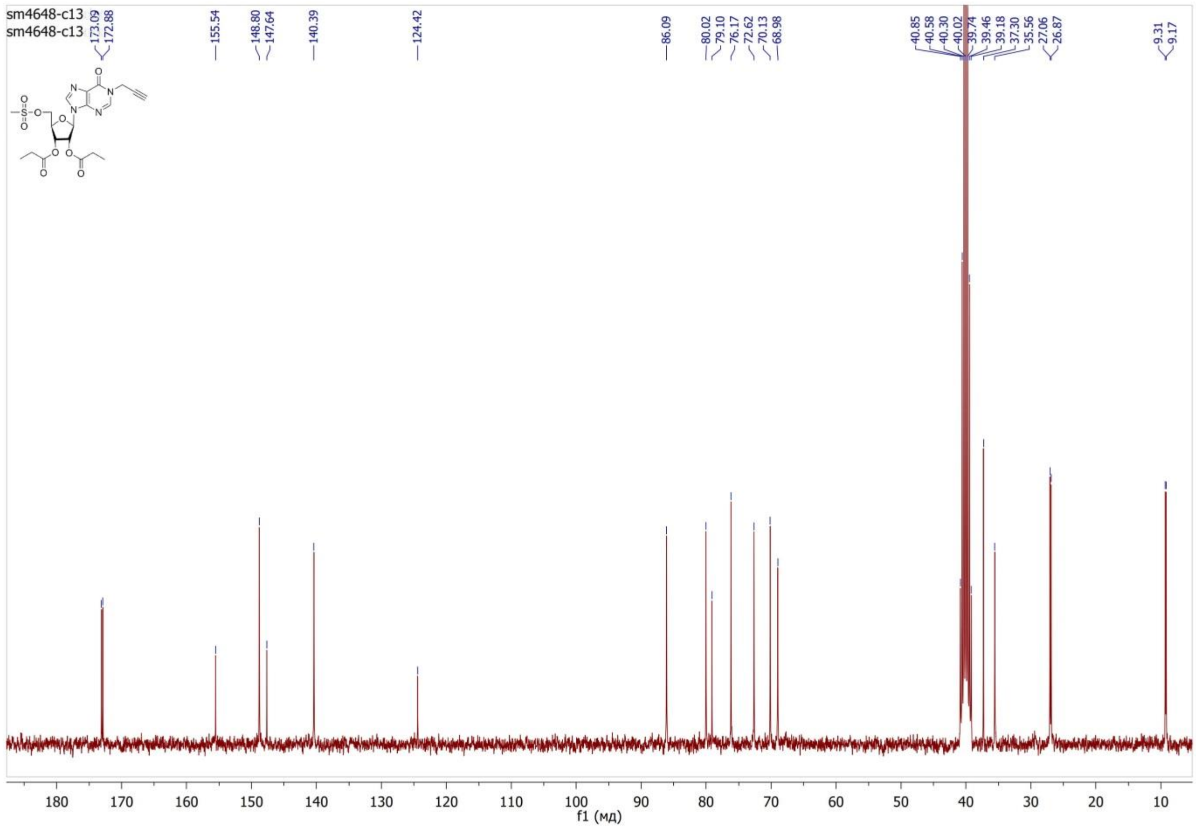
sm4648-c13
sm4648-c13
f1 (мд)

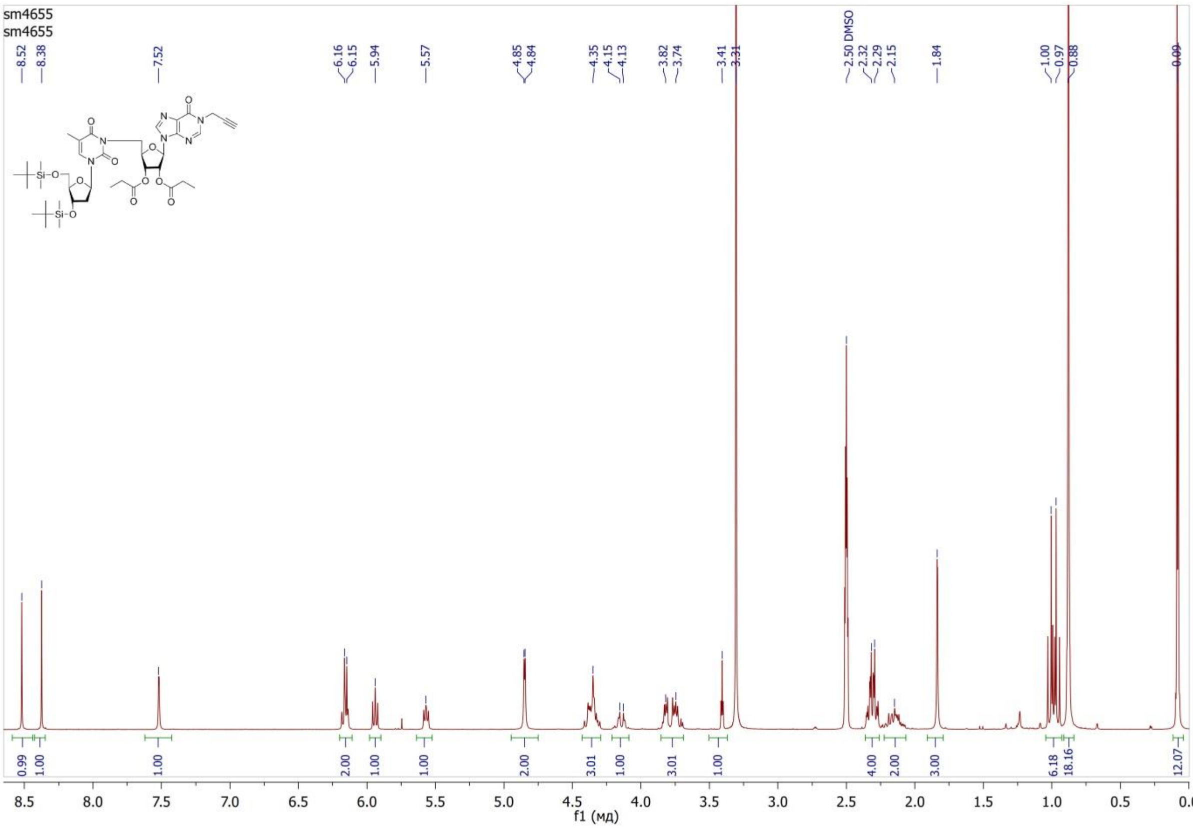
sm4655
sm4655
8.52
8.38
7.52
6.16
6.15
5.94
5.57
4.85
4.84
4.35
4.15
4.13
3.82
3.74
3.41
3.31
2.50 DMSO
2.32
2.29
2.15
1.84
1.00
0.97
0.88
0.09
0.99
1.00
1.00
2.00
1.00
1.00
2.00
3.01
1.00
3.01
1.00
4.00
2.00
3.00
6.18
18.16
12.07
f1 (мд)

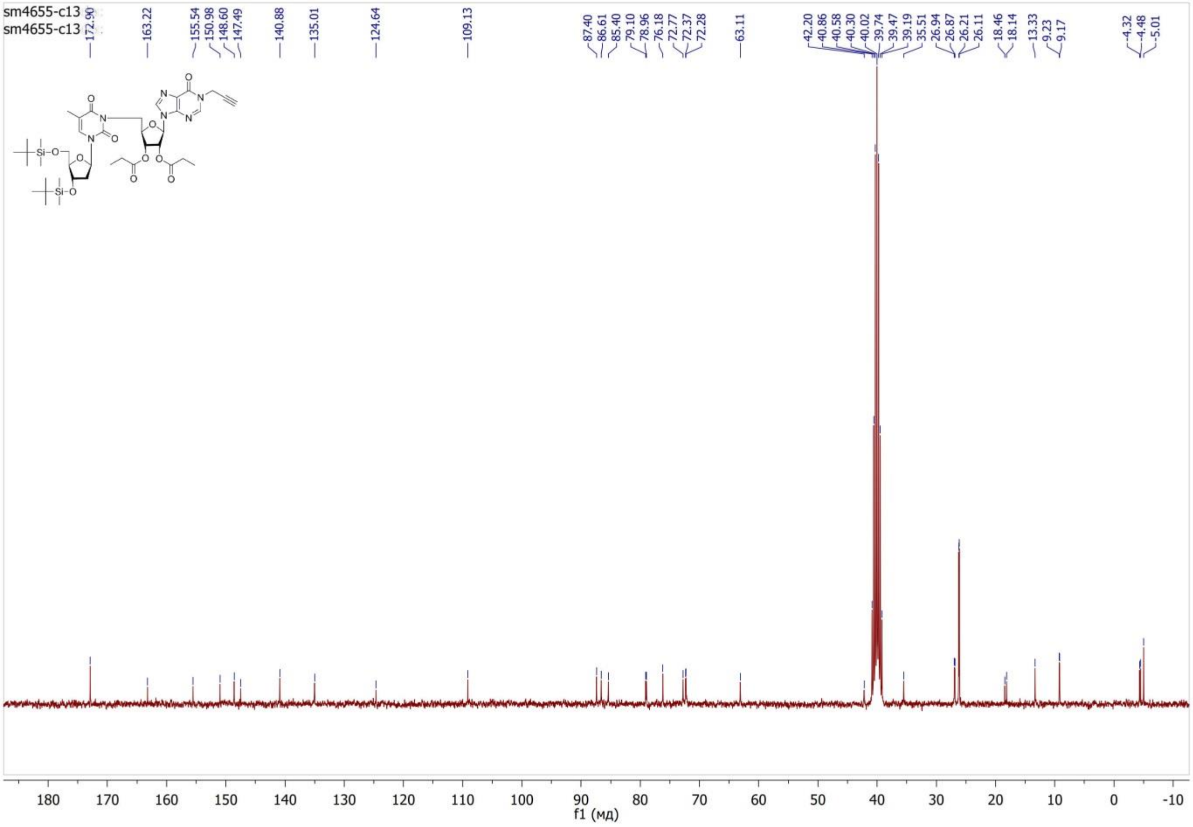
sm4655-c13
sm4655-c13
172.90
163.22
155.54
150.98
148.60
147.49
140.88
135.01
124.64
109.13
87.40
86.61
85.40
79.10
78.96
76.18
72.77
72.37
72.28
63.11
42.20
40.86
40.58
40.30
40.02
39.74
39.47
39.19
35.51
26.94
26.87
26.21
26.11
18.46
18.14
13.33
9.23
9.17
-4.32
-4.48
-5.01
f1 (мд)

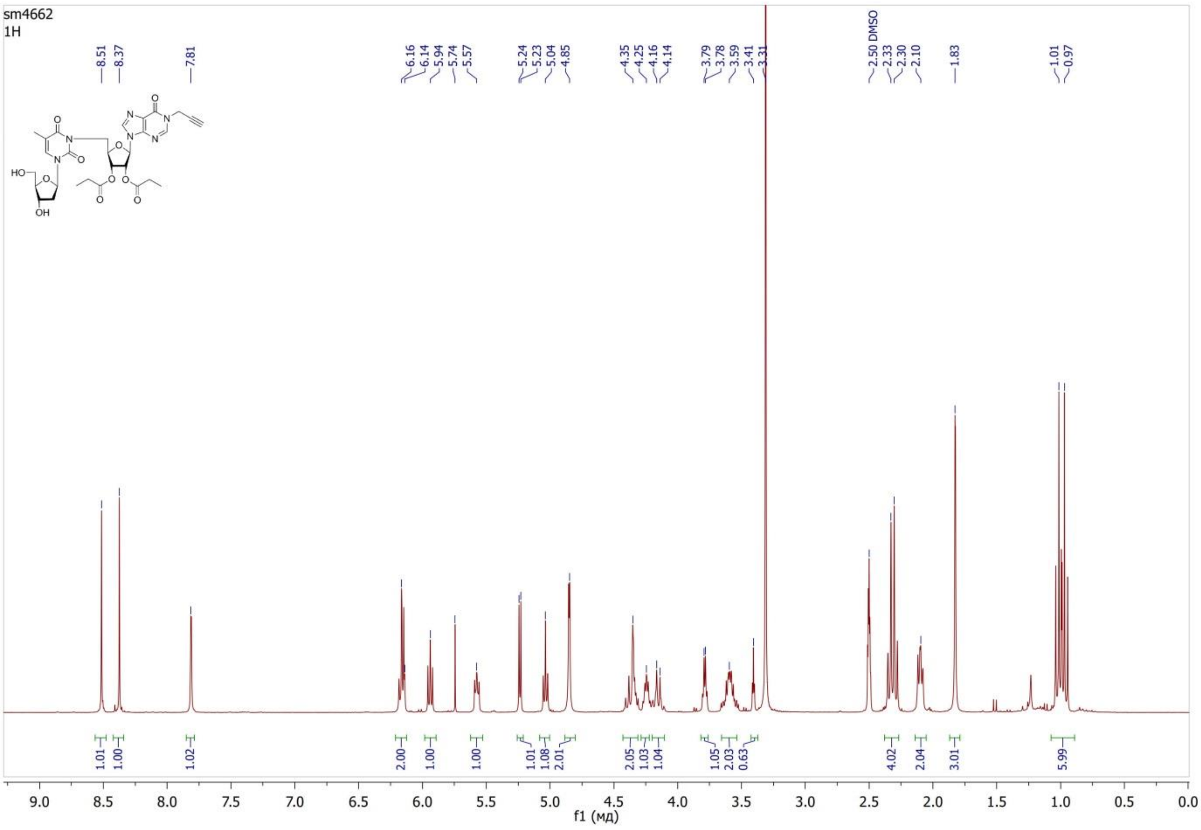
sm4662
1H
8.51
8.37
7.81
6.16
6.14
5.94
5.74
5.57
5.24
5.23
5.04
4.85
4.35
4.25
4.16
4.14
3.79
3.78
3.59
3.41
3.31
2.50 DMSO
2.33
2.30
2.10
1.83
1.01
0.97
1.01
1.00
1.02
2.00
1.00
1.00
1.01
1.08
2.01
2.05
1.03
1.04
1.05
2.03
0.63
4.02
2.04
3.01
5.99
9.0 8.5 8.0 7.5 7.0 6.5 6.0 5.5 5.0 4.5 4.0 3.5 3.0 2.5 2.0 1.5 1.0 0.5 0.0
f1 (мд)
HO
OH
O
N
N
O
O
O
O
O
O
N
N
N
N
O

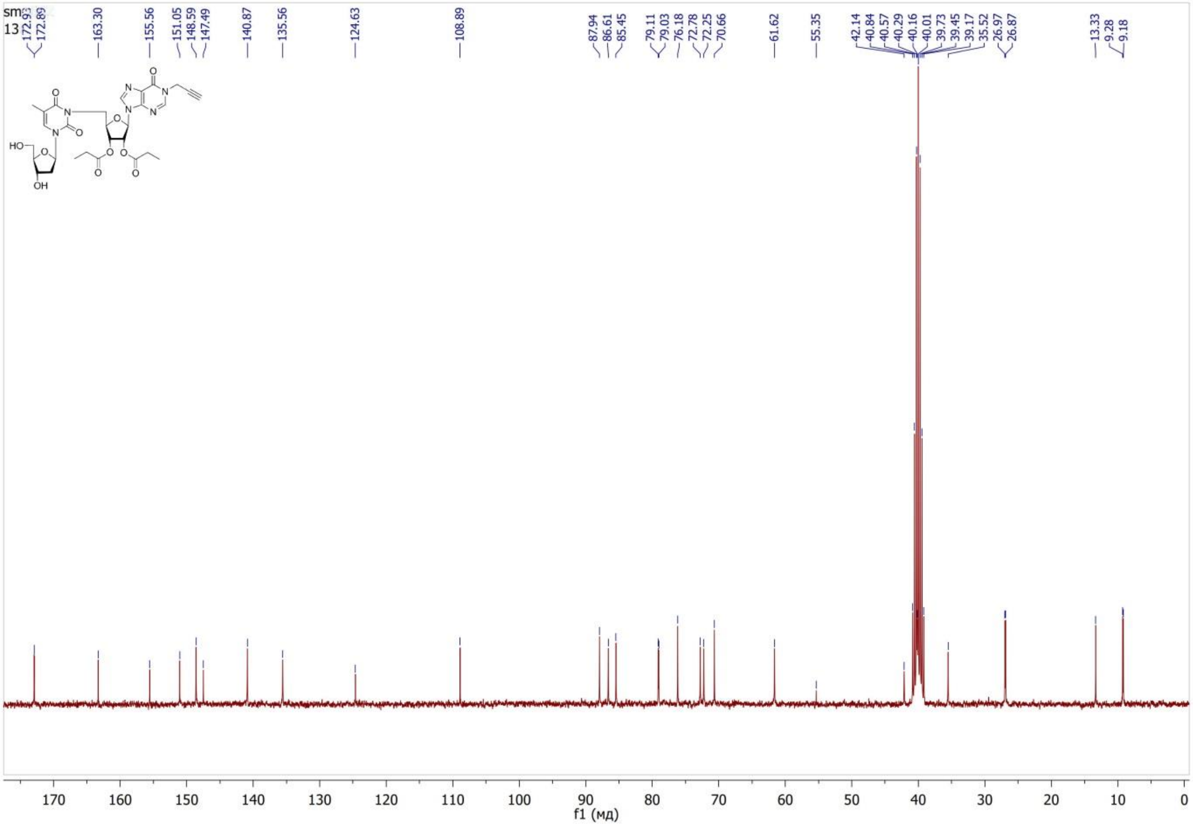
sm
13
172.93
172.89
163.30
155.56
151.05
148.59
147.49
140.87
135.56
124.63
108.89
87.94
86.61
85.45
79.11
79.03
76.18
72.78
72.25
70.66
61.62
55.35
42.14
40.84
40.57
40.29
40.16
40.01
39.73
39.45
39.17
35.52
26.97
26.87
13.33
9.28
9.18
170 160 150 140 130 120 110 100 90 80 70 60 50 40 30 20 10 0
f1 (мд)
HO
OH
O
N
N
O
O
O
O
O
O
N
N
N
N
O

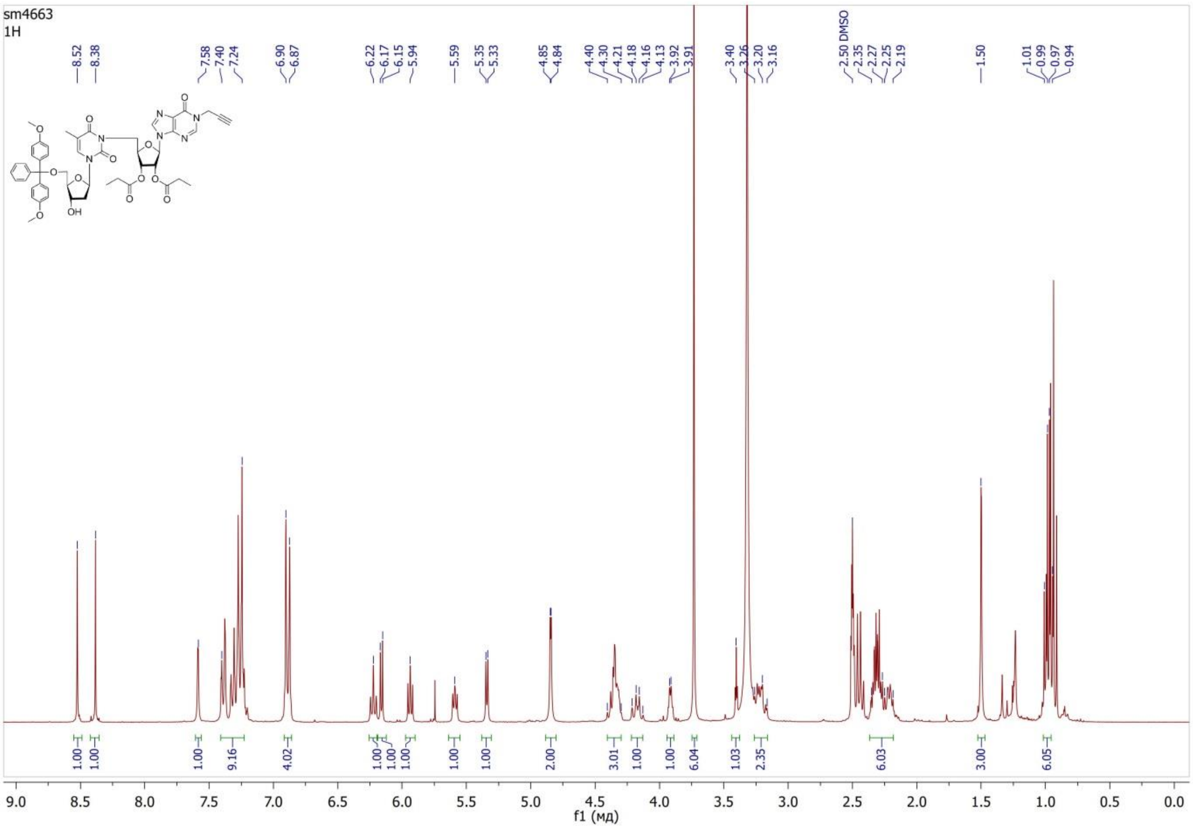
sm4663
1H
f1 (мд)

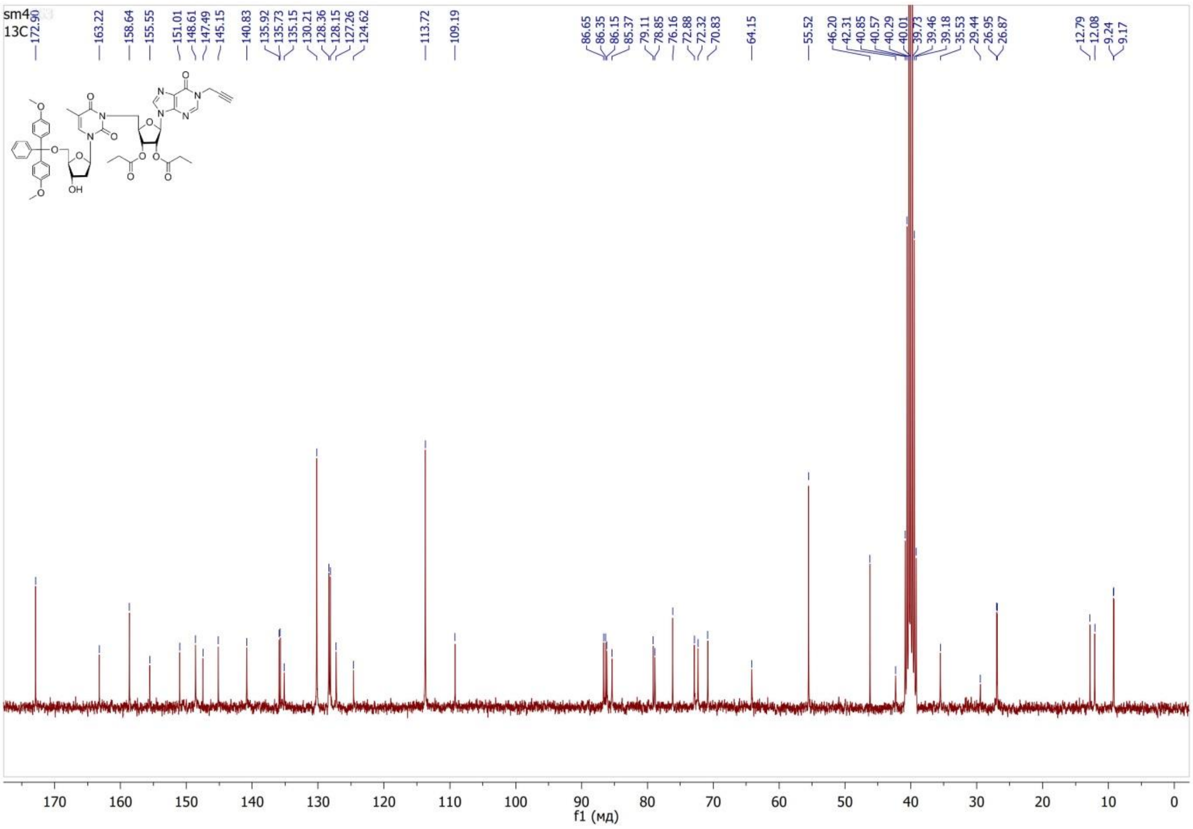
13C
f1 (мд)

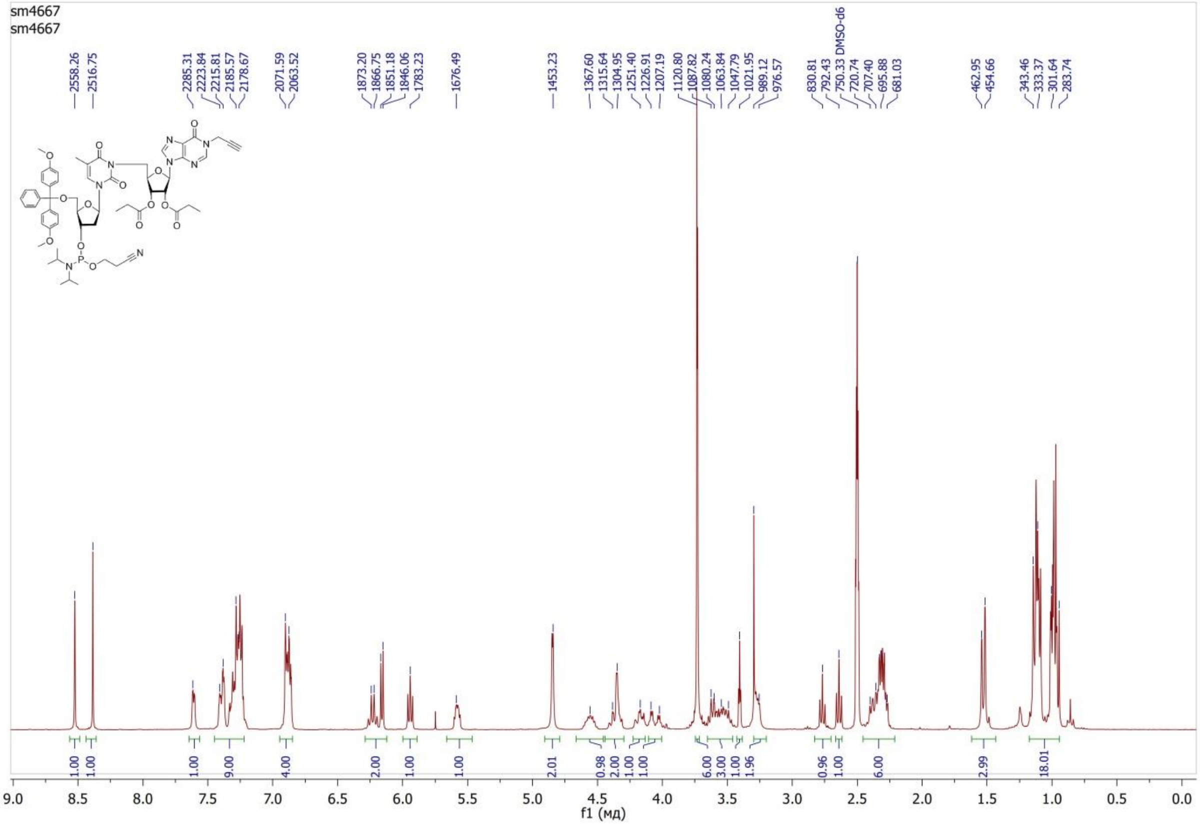
sm4667
sm4667
DMSO-d6
f1 (мд)

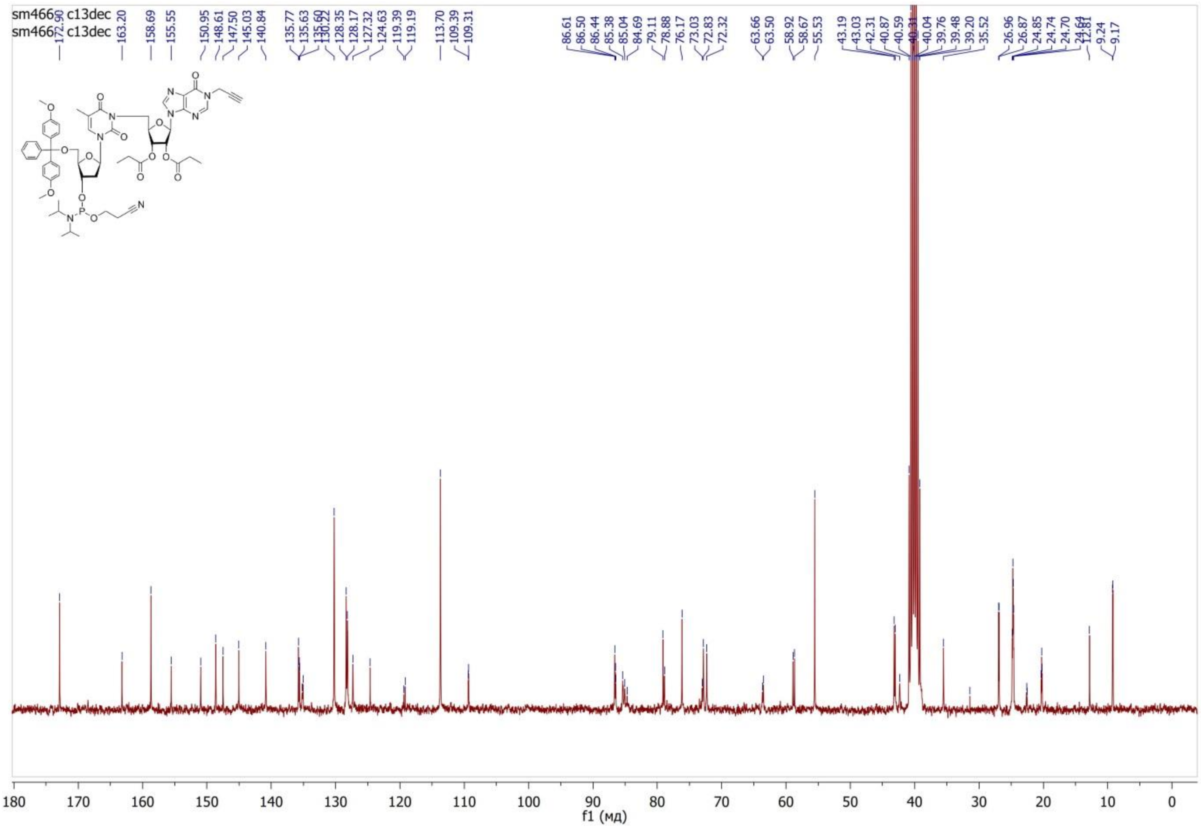
sm4667 c13dec
sm4667 c13dec
f1 (мд)

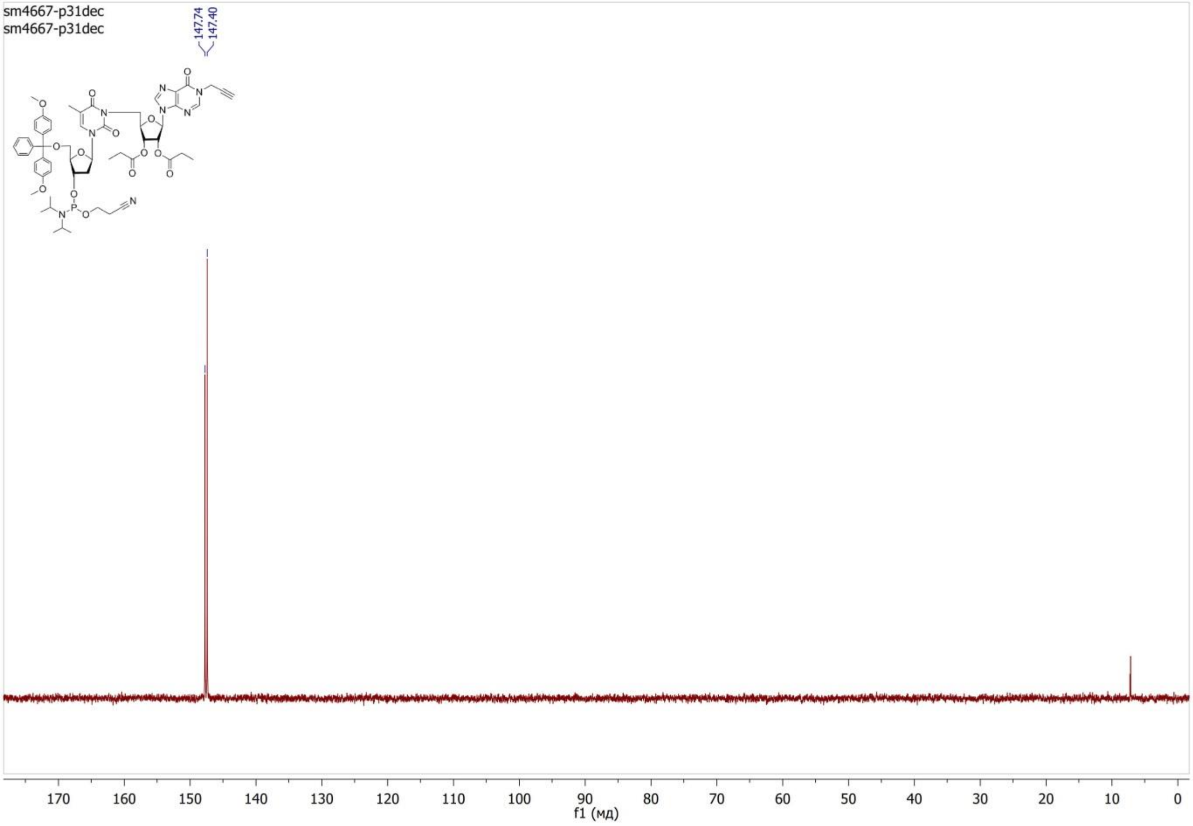
sm4667-p31dec
sm4667-p31dec
147.74
147.40
f1 (мд)

**HRMS spectra**

The following parameters were used: capillary voltage 4500 V; mass scanning range: m/z 50–3000; external calibration with Electrospray Calibrant Solution (Fluka, Darmstadt, Germany); gas pressure 0.4 bar; nitrogen spray gas (4 L/min); interface temperature: 180 °C; flow rate 3 µL/ min. Molecular and fragmentation ions in the spectra were analyzed and matched with the appropriately calculated *m/z* and isotopic profiles in the Bruker DataAnalysis 4.0 program. Earlier-prepared dry samples were dissolved in 50% acetonitrile in water and injected into the mass-spectrometer spray chamber from an Agilent 1260 HPLC chromatograph equipped with an Agilent Poroshell 120 EC-C18 column (3.0 × 50 mm; 2.7 µm; Germany) and a compatible pre-column cartridge using an autosampler. The column was eluted with a mixture of acetonitrile (A) and water (B) in a gradient concentration with the flow rate of 400 µL/min in the following gradient parameters: 0–6 min – 40%-85% A, 6–7.5 min – 85% A, 7.5–8 min – 85%-40% A, 8–10 min – 40% A.

| Compound## | Label | Brutto formula | *m/z*: calcd for $[M+H]^+$ | found |
|---|---|---|---|---|
| **1** | Smikh-222 (2) | $C_{26}H_{30}N_4O_5Si$ | 507.2058 | 507.2061 |
| **2** | Smikh-223 (3) | $C_{29}H_{32}N_4O_5Si$ | 545.2215 | 545.2211 |
| **3** | Smikh-224 (4) | $C_{35}H_{40}N_4O_7Si$ | 657.2739 | 657.2740 |
| **4** | Smikh-225 (5) | $C_{19}H_{22}N_4O_7$ | 419.1561 | 419.1561 |
| **5** | Smikh-226 (6) | $C_{20}H_{24}N_4O_9S$ | 497.1337 | 497.1336 |
| **6** | Smikh-227 (7) | $C_{41}H_{62}N_6O_{11}Si_2$ | 871.4088 | 871.4090 |
| **7** | Smikh-228 (8) | $C_{29}H_{34}N_6O_{11}$ | 643.2358 | 643.2358 |
| **8** | Smikh-229 (9) | $C_{50}H_{52}N_6O_{13}$ | 945.3665 | 945.3655 |
| **9** | Smikh-222 (10) | $C_{59}H_{69}N_8O_{14}P$ | 1145.4744 | 1145.4753 |

# Display Report

| **Analysis Info** | | | |
|---|---|---|---|
| Analysis Name | D:\Data\Mikh-222 (2)_2_01_4376.d | Acquisition Date | 11/14/2023 4:04:40 PM |
| Method | la_2.2_small_40acn3.m | Operator | BDAL@DE |
| Sample Name | Mikh-222 (2) | Instrument | compact 8255754.20088 |
| Comment | | | |

**Acquisition Parameter**

| | | | | | |
|---|---|---|---|---|---|
| Source Type | ESI | Ion Polarity | Positive | Set Nebulizer | 0.4 Bar |
| Focus | Active | Set Capillary | 4500 V | Set Dry Heater | 180 C |
| Scan Begin | 50 m/z | Set End Plate Offset | -500 V | Set Dry Gas | 4.0 l/min |
| Scan End | 3000 m/z | Set Charging Voltage | 2000 V | Set Divert Valve | Source |
| | | Set Corona | 0 nA | Set APCI Heater | 0 C |

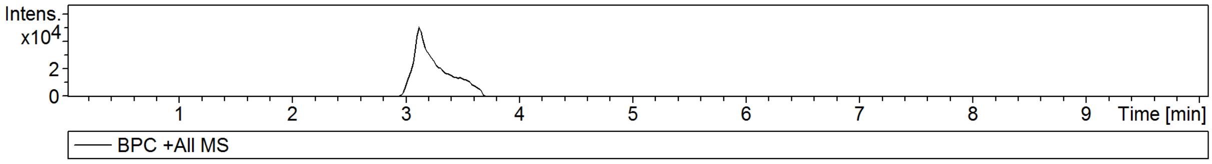


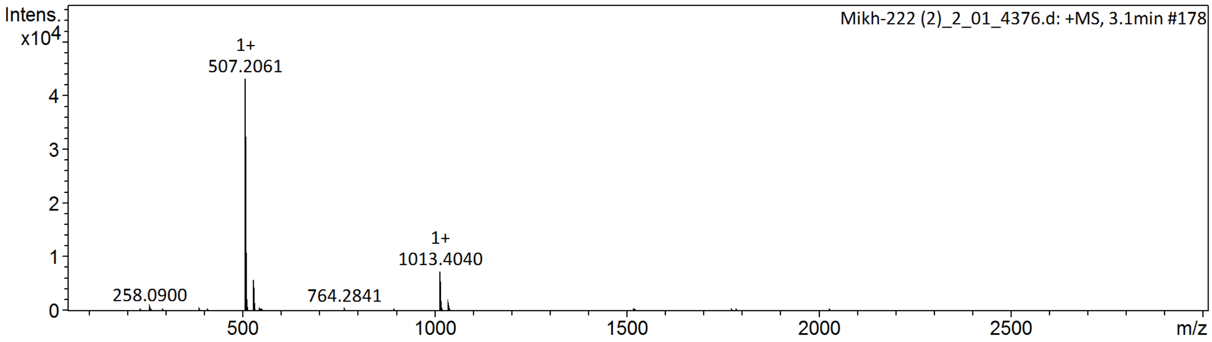


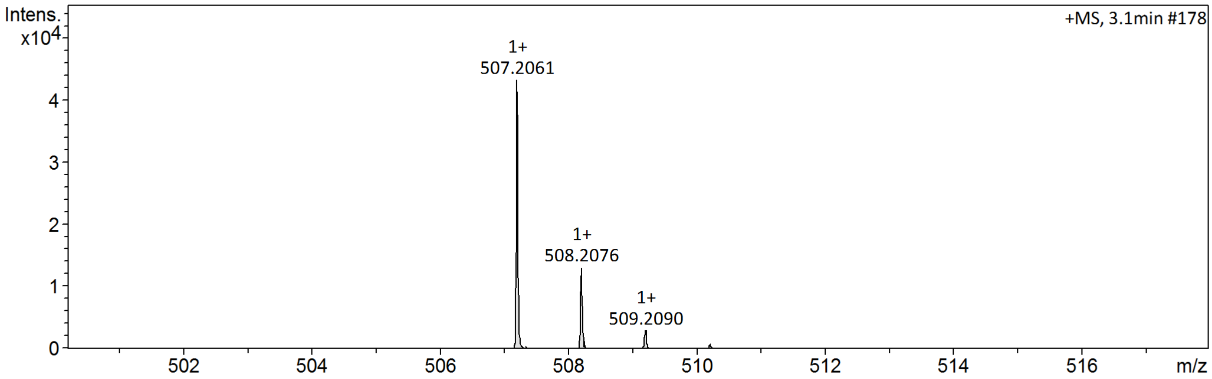


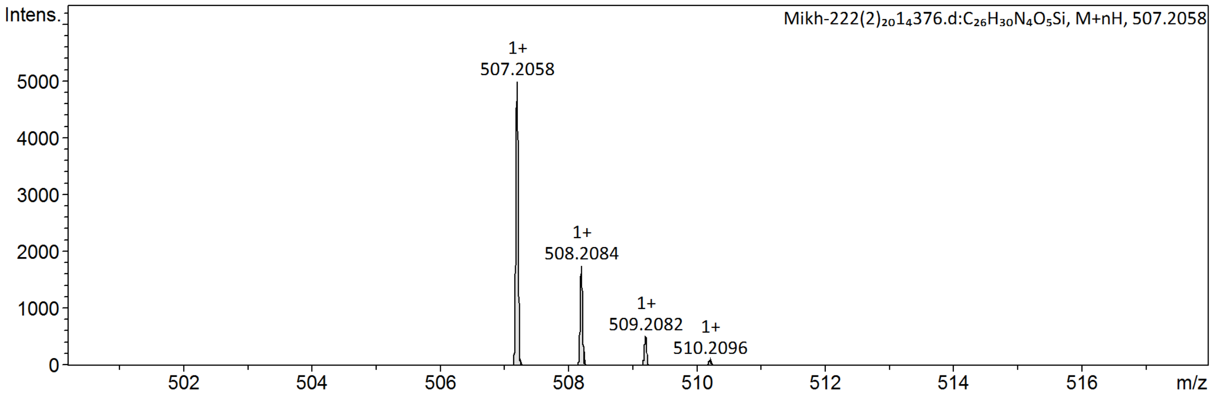

# Display Report

**Analysis Info**

Acquisition Date 11/14/2023 4:16:20 PM

| | |
|---|---|
| Analysis Name | D:\Data\Mikh-223 (3)_3_01_4377.d |
| Method | la_2.2_small_40acn3.m |
| Sample Name | Mikh-223 (3) |
| Comment | |

Operator BDAL@DE
Instrument compact 8255754.20088

**Acquisition Parameter**

| | | | | | |
|---|---|---|---|---|---|
| Source Type | ESI | Ion Polarity | Positive | Set Nebulizer | 0.4 Bar |
| Focus | Active | Set Capillary | 4500 V | Set Dry Heater | 180 C |
| Scan Begin | 50 m/z | Set End Plate Offset | -500 V | Set Dry Gas | 4.0 l/min |
| Scan End | 3000 m/z | Set Charging Voltage | 2000 V | Set Divert Valve | Source |
| | | Set Corona | 0 nA | Set APCI Heater | 0 C |

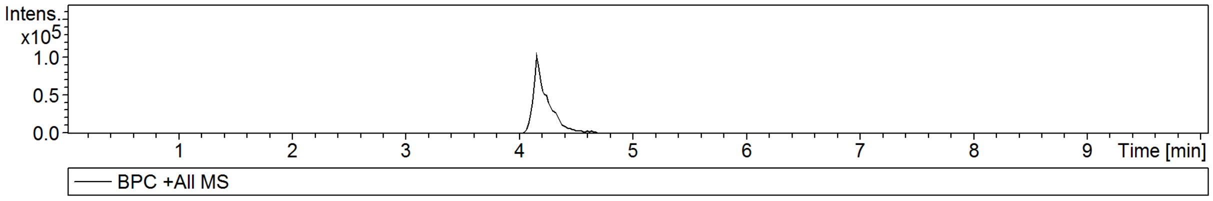


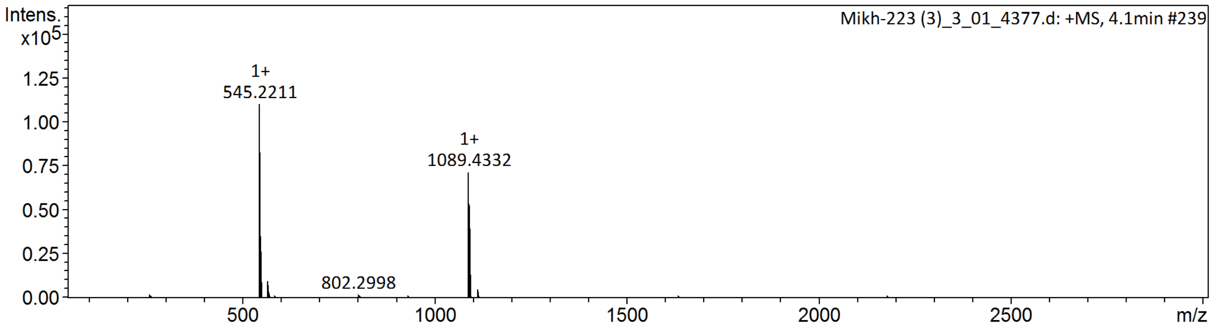


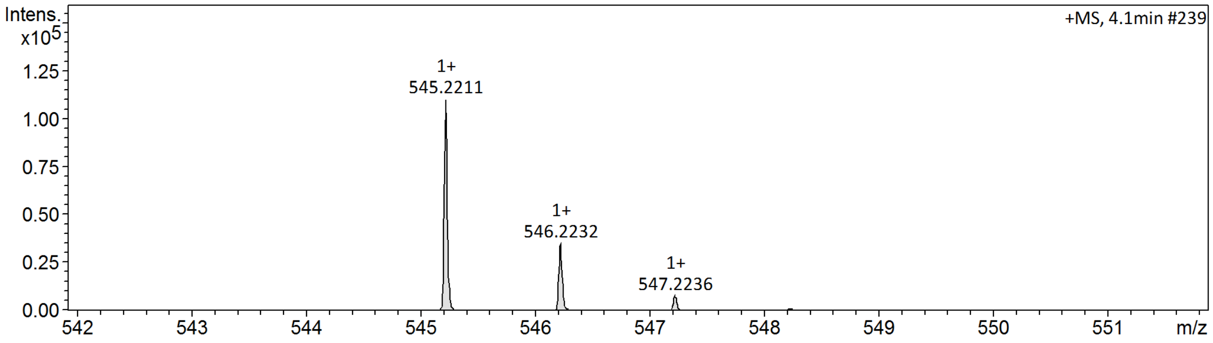


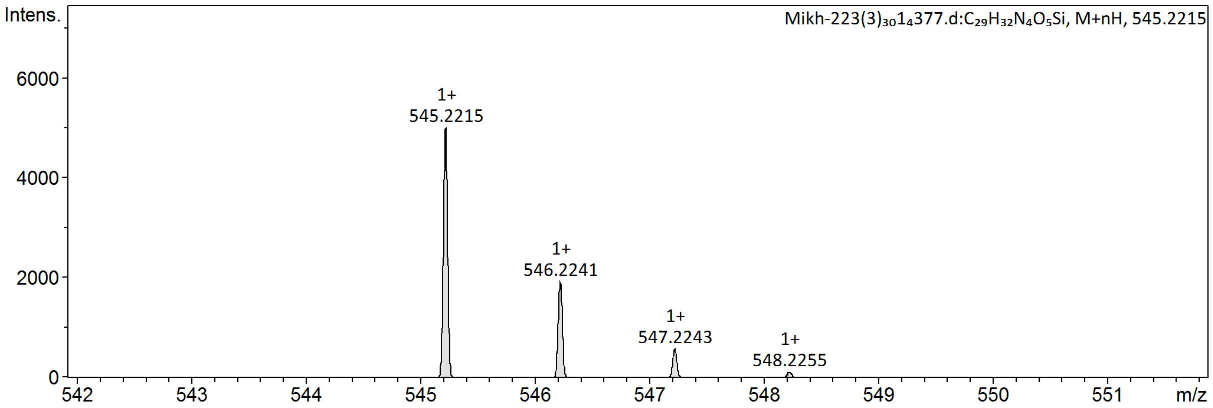

# Display Report

**Analysis Info**

| | | | |
|---|---|---|---|
| Analysis Name | D:\Data\Mikh-224 (4)_4_01_4378.d | Acquisition Date | 11/14/2023 4:27:55 PM |
| Method | la_2.2_small_40acn3.m | Operator | BDAL@DE |
| Sample Name | Mikh-224 (4) | Instrument | compact 8255754.20088 |
| Comment | | | |

**Acquisition Parameter**

| | | | | | |
|---|---|---|---|---|---|
| Source Type | ESI | Ion Polarity | Positive | Set Nebulizer | 0.4 Bar |
| Focus | Active | Set Capillary | 4500 V | Set Dry Heater | 180 C |
| Scan Begin | 50 m/z | Set End Plate Offset | -500 V | Set Dry Gas | 4.0 l/min |
| Scan End | 3000 m/z | Set Charging Voltage | 2000 V | Set Divert Valve | Source |
| | | Set Corona | 0 nA | Set APCI Heater | 0 C |

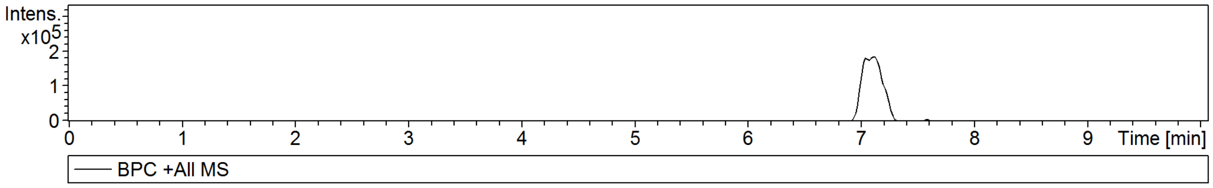


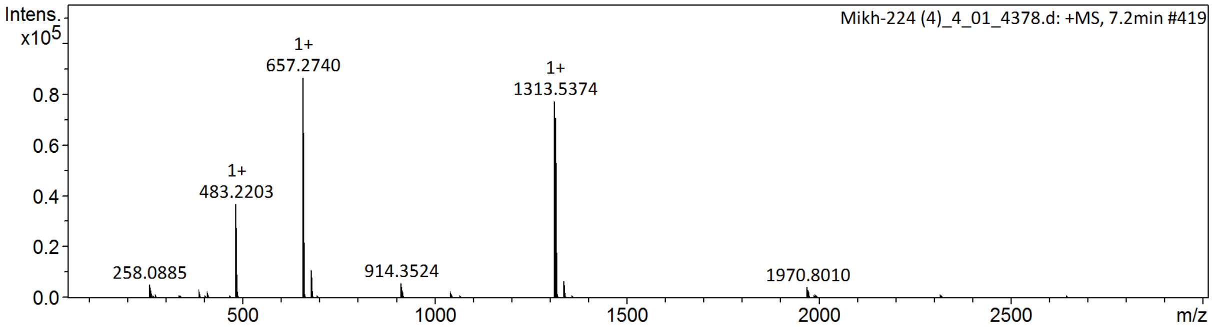


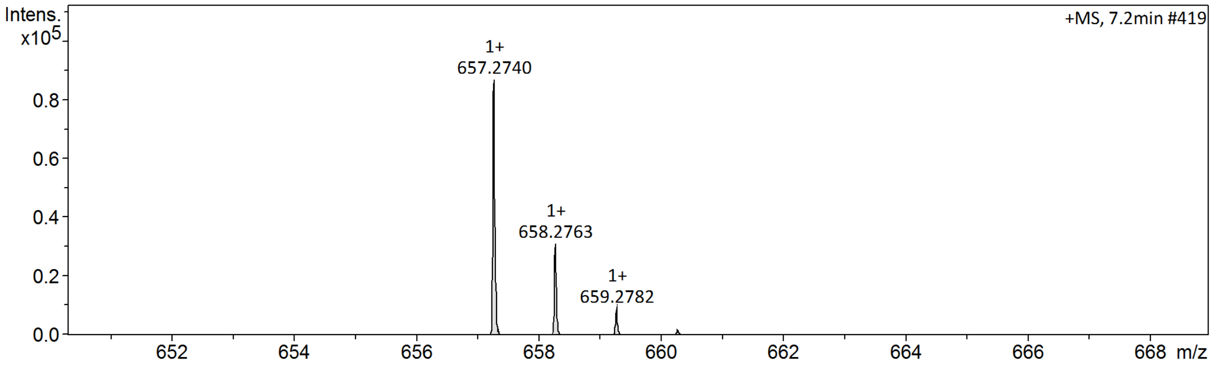


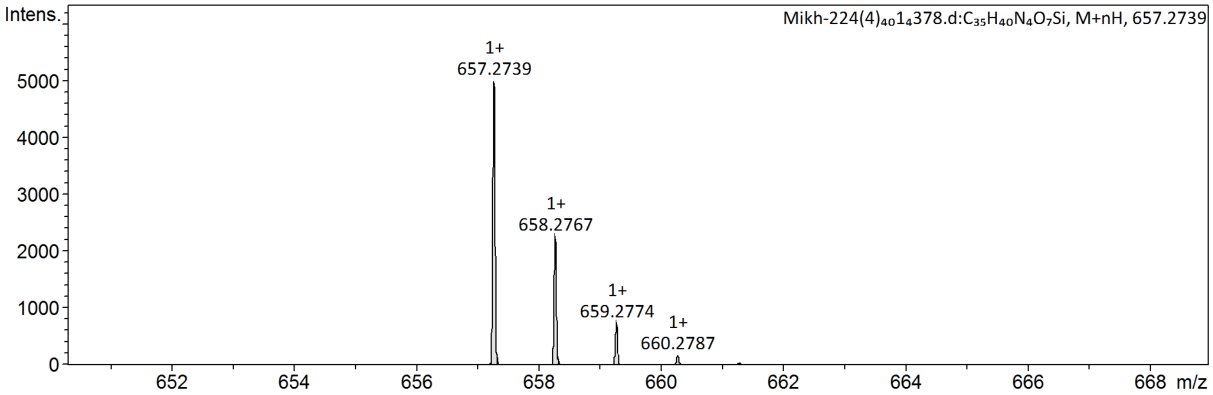

# Display Report

**Analysis Info**

| | |
|---|---|
| Analysis Name | D:\Data\Mikh-225 (5)_5_01_4379.d |
| Method | la_2.2_small_40acn3.m |
| Sample Name | Mikh-225 (5) |
| Comment | |
| Acquisition Date | 11/14/2023 4:39:28 PM |
| Operator | BDAL@DE |
| Instrument | compact 8255754.20088 |

**Acquisition Parameter**

| | | | | | |
|---|---|---|---|---|---|
| Source Type | ESI | Ion Polarity | Positive | Set Nebulizer | 0.4 Bar |
| Focus | Active | Set Capillary | 4500 V | Set Dry Heater | 180 C |
| Scan Begin | 50 m/z | Set End Plate Offset | -500 V | Set Dry Gas | 4.0 l/min |
| Scan End | 3000 m/z | Set Charging Voltage | 2000 V | Set Divert Valve | Source |
| | | Set Corona | 0 nA | Set APCI Heater | 0 C |

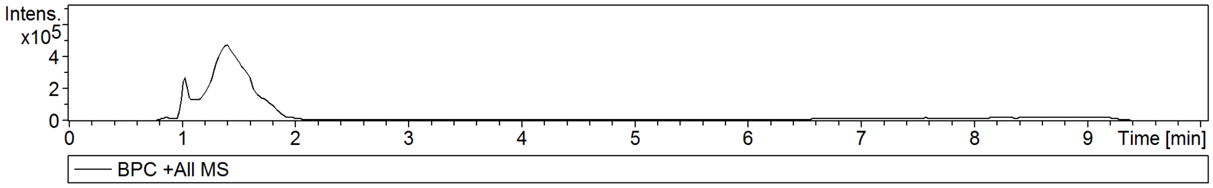


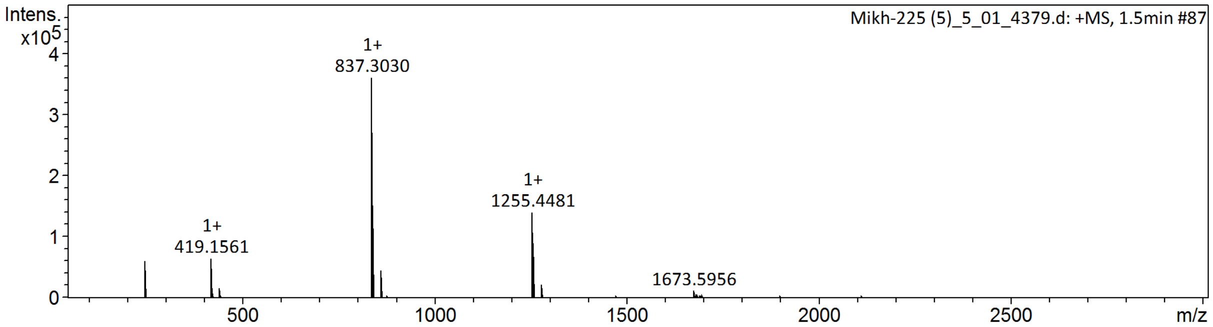


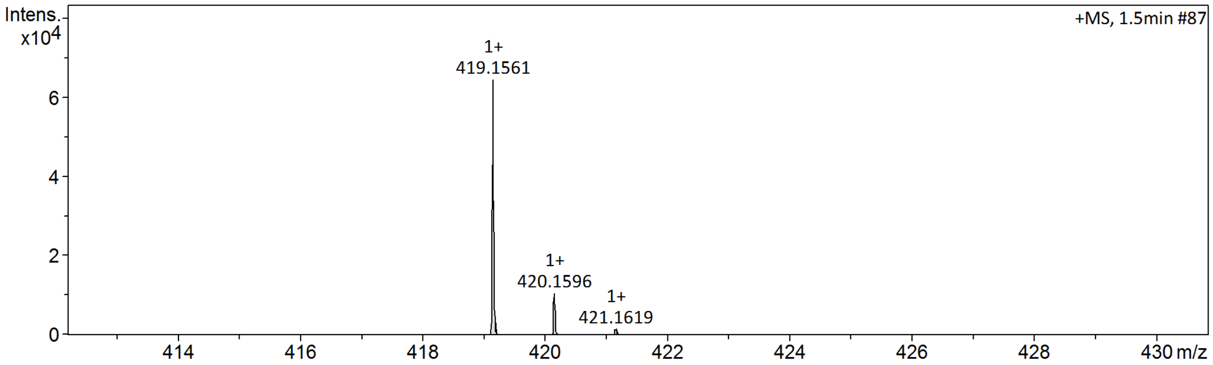


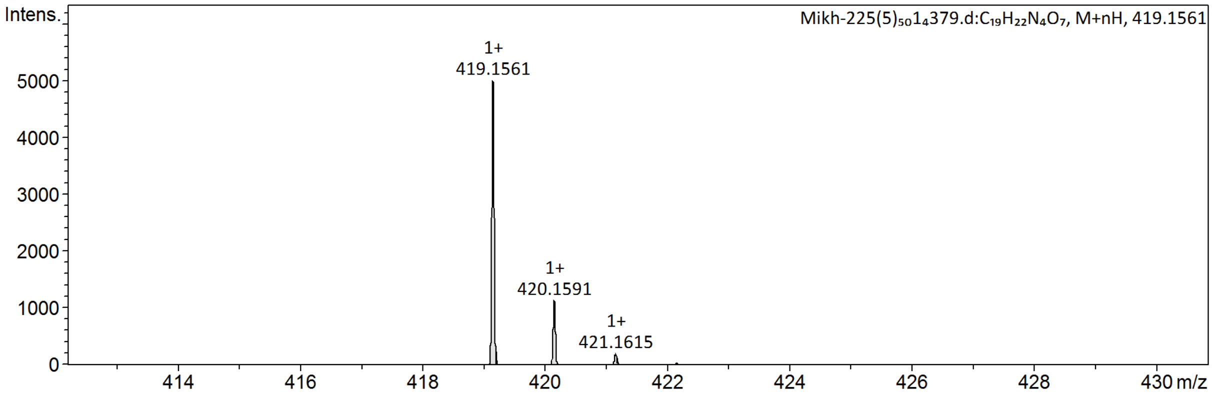

# Display Report

**Analysis Info**

| | | | | |
|---|---|---|---|---|
| Analysis Name | D:\Data\Mikh-226 (6)_6_01_4380.d | Acquisition Date | 11/14/2023 4:51:01 PM | |
| Method | la_2.2_small_40acn3.m | Operator | BDAL@DE | |
| Sample Name | Mikh-226 (6) | Instrument | compact | 8255754.20088 |
| Comment | | | | |

**Acquisition Parameter**

| | | | | | |
|---|---|---|---|---|---|
| Source Type | ESI | Ion Polarity | Positive | Set Nebulizer | 0.4 Bar |
| Focus | Active | Set Capillary | 4500 V | Set Dry Heater | 180 C |
| Scan Begin | 50 m/z | Set End Plate Offset | -500 V | Set Dry Gas | 4.0 l/min |
| Scan End | 3000 m/z | Set Charging Voltage | 2000 V | Set Divert Valve | Source |
| | | Set Corona | 0 nA | Set APCI Heater | 0 C |

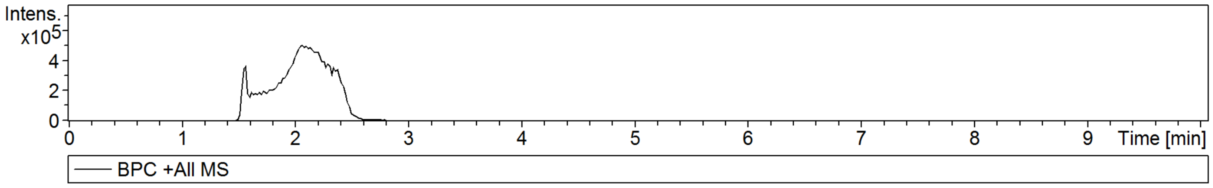


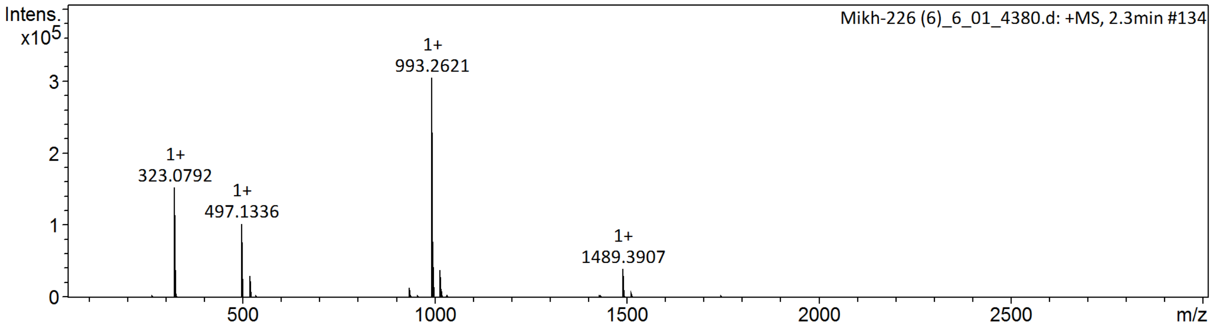


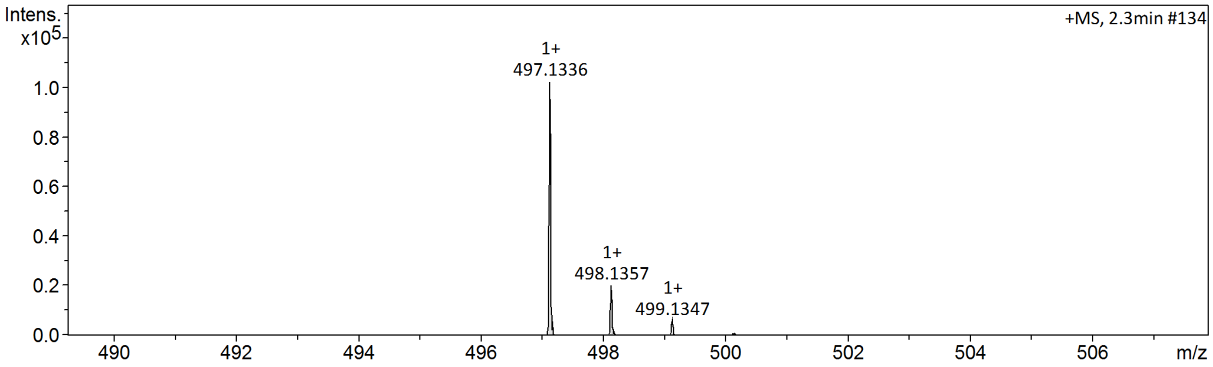


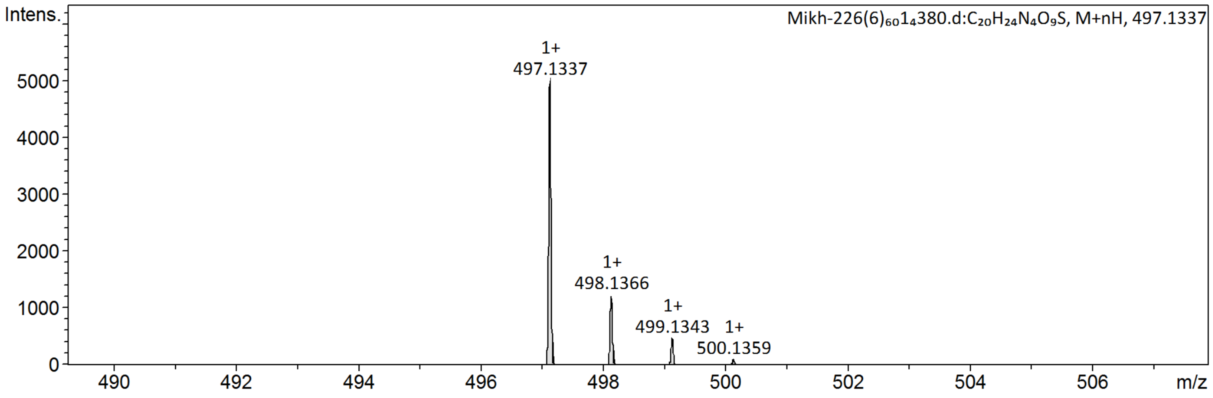

# Display Report

**Analysis Info**

| | | | |
|---|---|---|---|
| Analysis Name | D:\Data\Empty-Mikh-227 (7)-2_91_01_4387.d | Acquisition Date | 11/23/2023 9:32:14 PM |
| Method | la_2.2_small_40acn3.m | Operator | BDAL@DE |
| Sample Name | Empty-Mikh-227 (7)-2 | Instrument | compact 8255754.20088 |
| Comment | | | |

**Acquisition Parameter**

| | | | | | |
|---|---|---|---|---|---|
| Source Type | ESI | Ion Polarity | Positive | Set Nebulizer | 0.4 Bar |
| Focus | Active | Set Capillary | 4500 V | Set Dry Heater | 180 C |
| Scan Begin | 50 m/z | Set End Plate Offset | -500 V | Set Dry Gas | 4.0 l/min |
| Scan End | 3000 m/z | Set Charging Voltage | 2000 V | Set Divert Valve | Source |
| | | Set Corona | 0 nA | Set APCI Heater | 0 C |

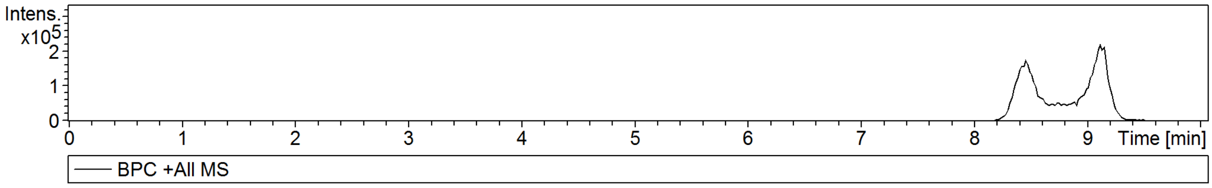


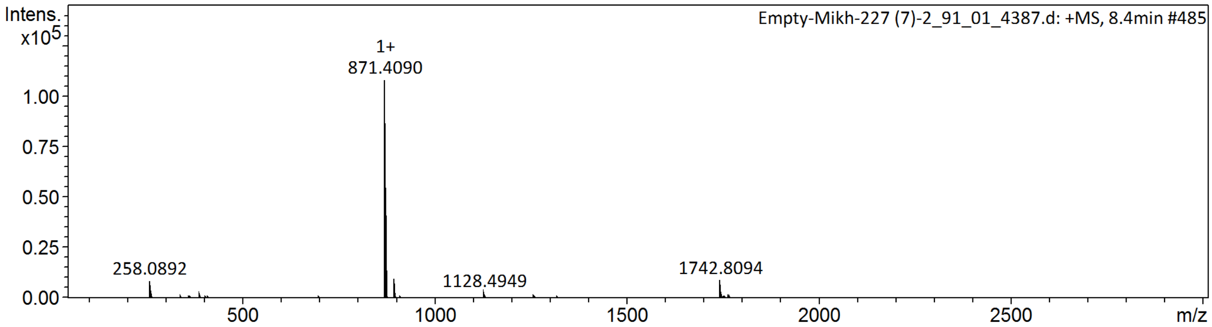


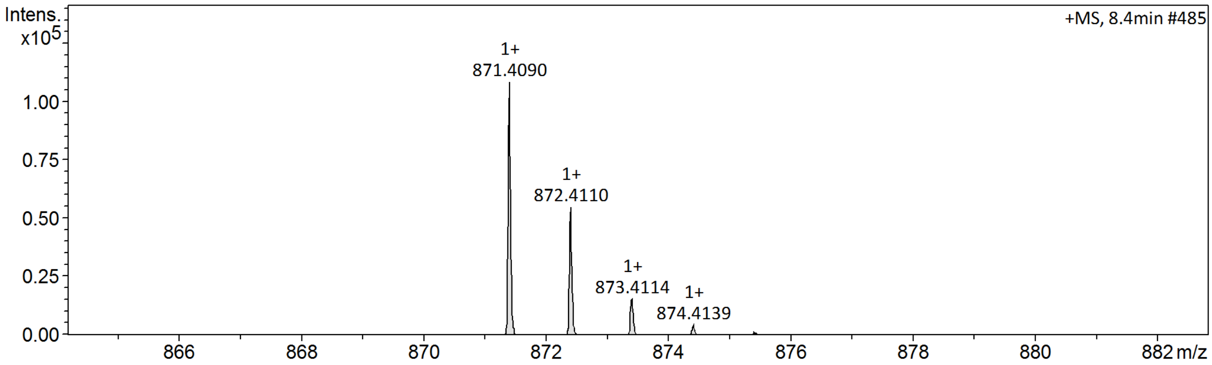


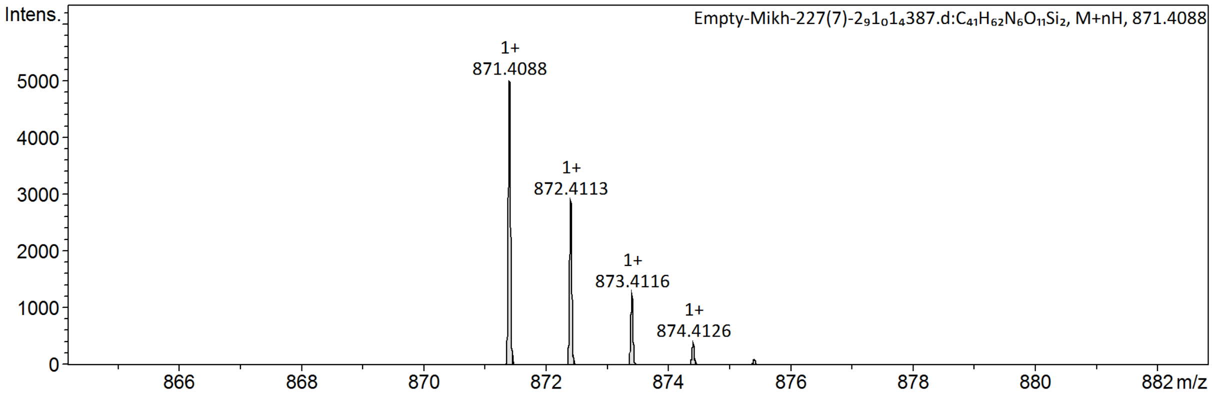

# Display Report

**Analysis Info**

| | |
|---|---|
| Analysis Name | D:\Data\Mikh-228 (8)_8_01_4382.d |
| Method | la_2.2_small_40acn3.m |
| Sample Name | Mikh-228 (8) |
| Comment | |

| | |
|---|---|
| Acquisition Date | 11/14/2023 5:14:23 PM |
| Operator | BDAL@DE |
| Instrument | compact 8255754.20088 |

**Acquisition Parameter**

| | | | | | |
|---|---|---|---|---|---|
| Source Type | ESI | Ion Polarity | Positive | Set Nebulizer | 0.4 Bar |
| Focus | Active | Set Capillary | 4500 V | Set Dry Heater | 180 C |
| Scan Begin | 50 m/z | Set End Plate Offset | -500 V | Set Dry Gas | 4.0 l/min |
| Scan End | 3000 m/z | Set Charging Voltage | 2000 V | Set Divert Valve | Source |
| | | Set Corona | 0 nA | Set APCI Heater | 0 C |

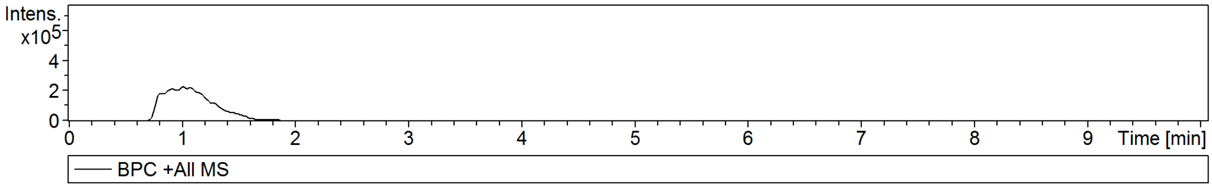


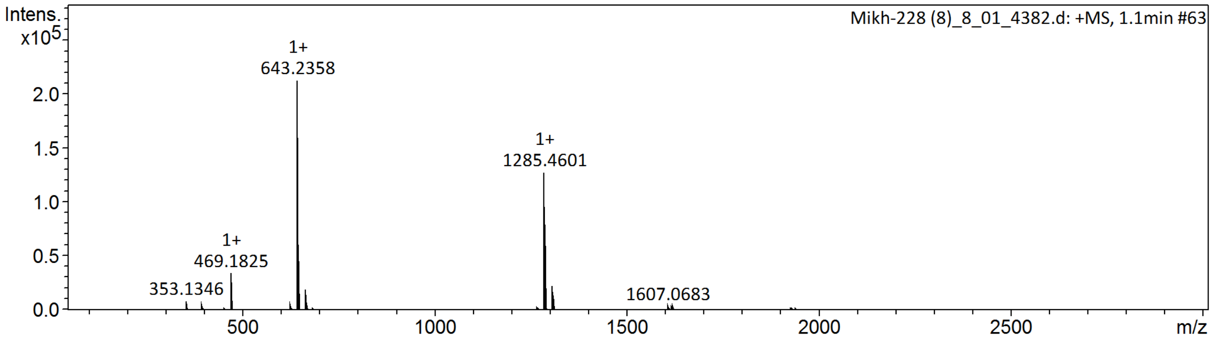


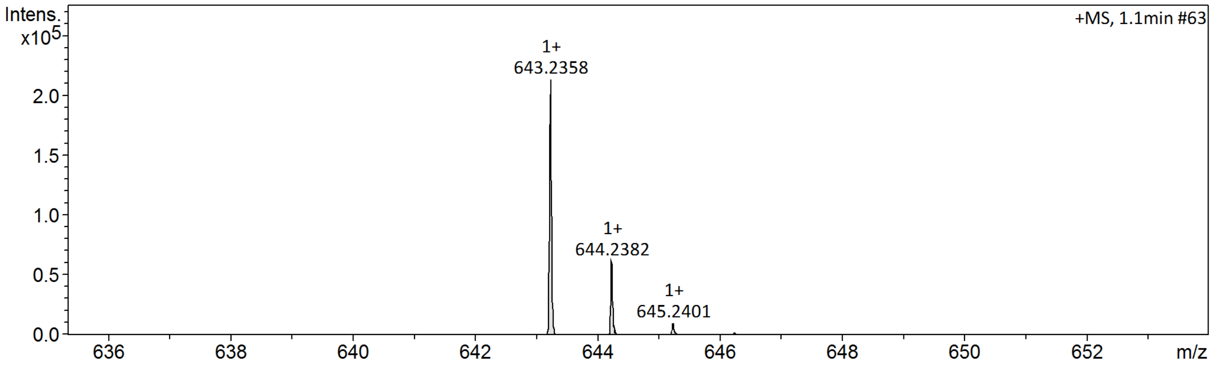


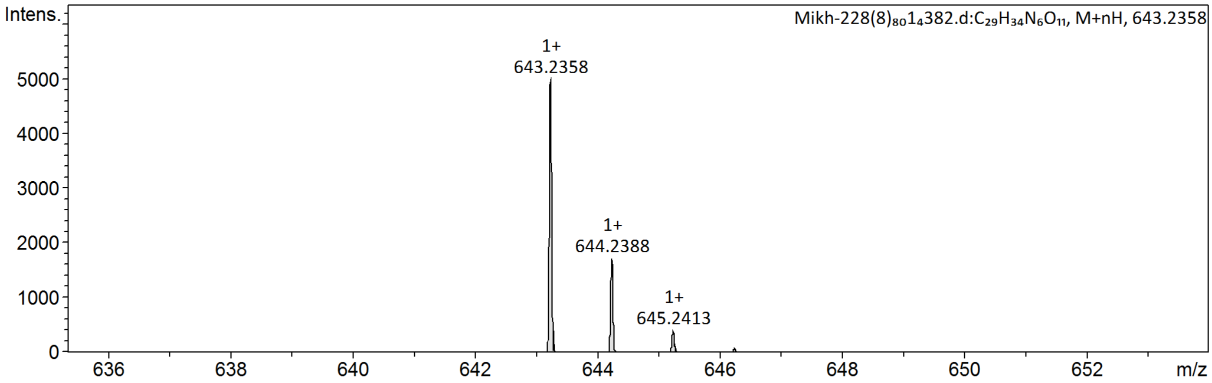

# Display Report

**Analysis Info**

| | | | |
|---|---|---|---|
| Analysis Name | D:\Data\Mikh-229 (9)_9_01_4383.d | Acquisition Date | 11/14/2023 5:26:02 PM |
| Method | la_2.2_small_40acn3.m | Operator | BDAL@DE |
| Sample Name | Mikh-229 (9) | Instrument | compact 8255754.20088 |
| Comment | | | |

**Acquisition Parameter**

| | | | | | |
|---|---|---|---|---|---|
| Source Type | ESI | Ion Polarity | Positive | Set Nebulizer | 0.4 Bar |
| Focus | Active | Set Capillary | 4500 V | Set Dry Heater | 180 C |
| Scan Begin | 50 m/z | Set End Plate Offset | -500 V | Set Dry Gas | 4.0 l/min |
| Scan End | 3000 m/z | Set Charging Voltage | 2000 V | Set Divert Valve | Source |
| | | Set Corona | 0 nA | Set APCI Heater | 0 C |

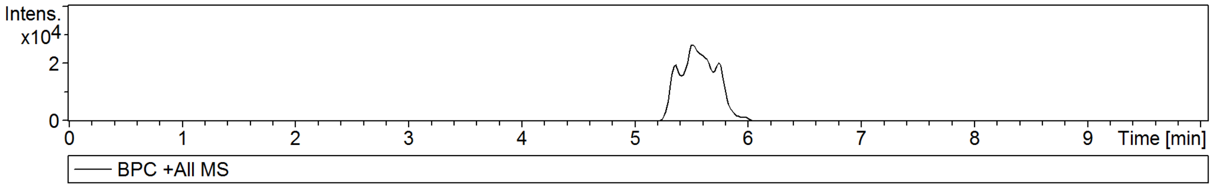


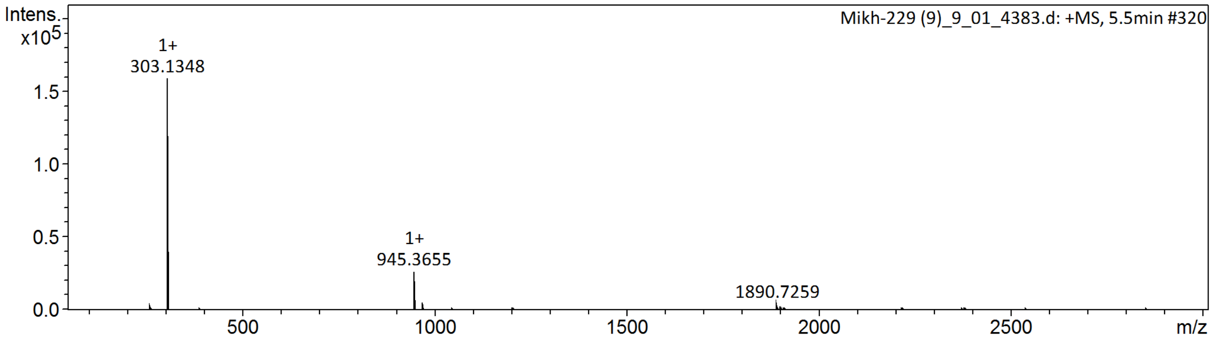


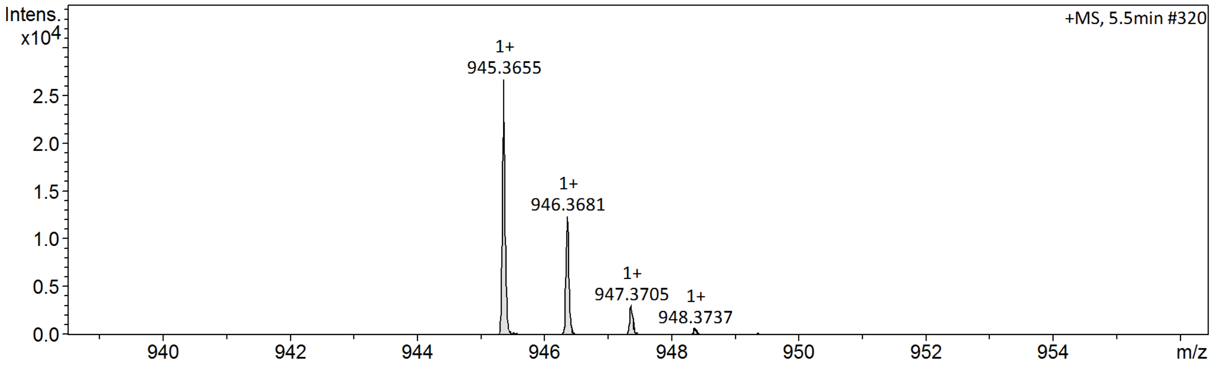


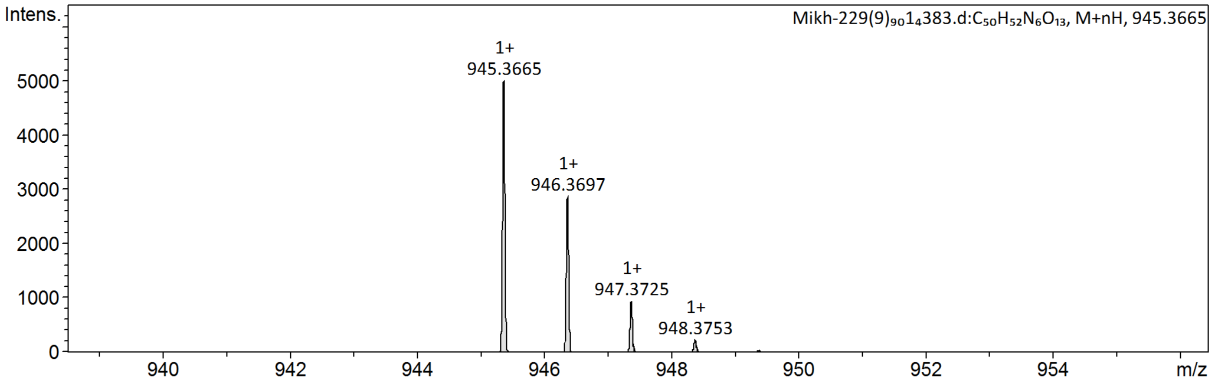

# Display Report

| **Analysis Info** | | | |
|---|---|---|---|
| Analysis Name | D:\Data\Mikh-230 (10)_10_01_4384.d | Acquisition Date | 11/14/2023 5:37:42 PM |
| Method | la_2.2_small_40acn3.m | Operator | BDAL@DE |
| Sample Name | Mikh-230 (10) | Instrument | compact 8255754.20088 |
| Comment | | | |

**Acquisition Parameter**

| | | | | | |
|---|---|---|---|---|---|
| Source Type | ESI | Ion Polarity | Positive | Set Nebulizer | 0.4 Bar |
| Focus | Active | Set Capillary | 4500 V | Set Dry Heater | 180 C |
| Scan Begin | 50 m/z | Set End Plate Offset | -500 V | Set Dry Gas | 4.0 l/min |
| Scan End | 3000 m/z | Set Charging Voltage | 2000 V | Set Divert Valve | Source |
| | | Set Corona | 0 nA | Set APCI Heater | 0 C |

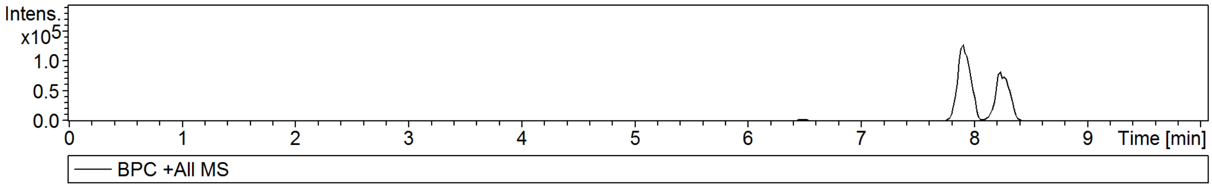


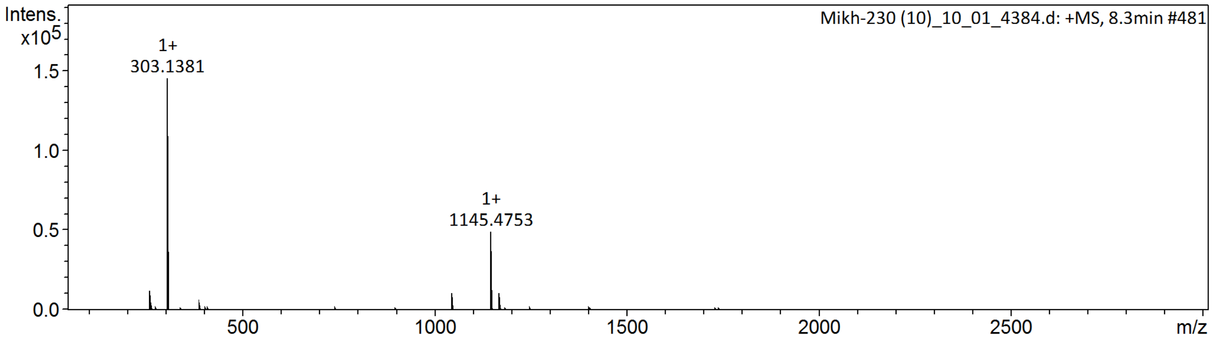


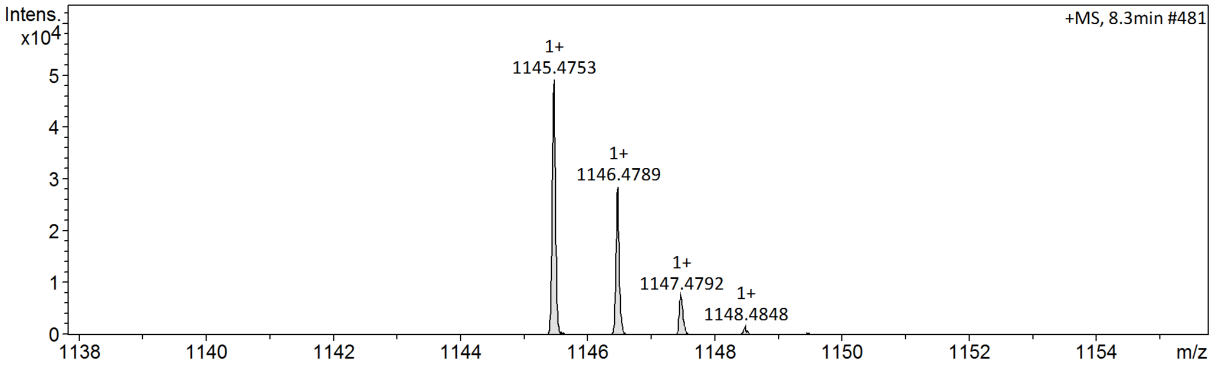


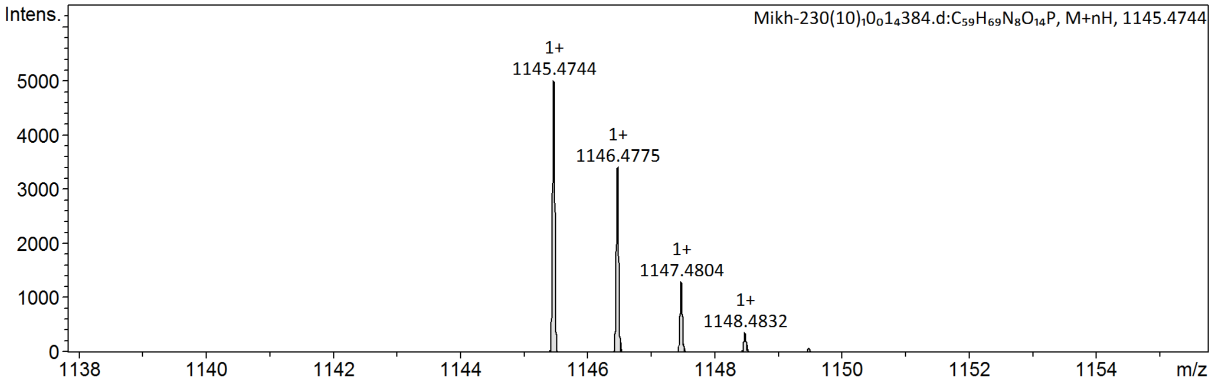

Supplement: Supplementary file 1 [file molecules-30-03769-s001.zip › molecules-3865303-supplementary.pdf]
